# Layering contrasting photoselective filters improves the simulation of foliar shade

**DOI:** 10.1186/s13007-022-00844-8

**Published:** 2022-02-08

**Authors:** Dominic P. Petrella, Florence Breuillin-Sessoms, Eric Watkins

**Affiliations:** Department of Horticultural Science, Univ. of Minnesota, 1970 Folwell Ave., St. Paul, MN 55108 USA

**Keywords:** Photoselective filter, Foliar shade, Spectral photon distribution, R:FR ratio, B:G ratio, Simulation

## Abstract

**Background:**

Neutral density shade cloth is commonly used for simulating foliar shade, in which it reduces light intensity without altering spectral quality. However, foliar shade also alters spectral quality, reducing the ratio of red to far-red (R:FR) light, altering the ratio of blue to green (B:G) light, and reducing ultraviolet light. Unlike shade cloth, photoselective filters can alter spectral quality, but the filters used in previous literature have not simulated foliar shade well. We examined the spectral quality of sunlight under color temperature blue (CTB), plus green (PG), and neutral density (ND) filters from LEE Filters, Rosco e-colour + and Cinegel brands either alone or layered, hypothesizing that the contrasting filter qualities would improve simulations. As a proof-of-concept, we collected spectral data under foliar shade to compare to data collected under photoselective filters.

**Results:**

Under foliar shade reductions in the R:FR ratio ranged from 0.11 to 0.54 (~ 1.18 in full sun), while reductions in the B:G ratio were as low as 0.53 in deep shade, or were as high as 1.11 in moderate shade (~ 0.87 in full sun). Neutral density filters led to near-neutral reductions in photosynthetically active radiation and reduced the R:FR ratio similar to foliar shade. Color temperature blue filters simulated the increased B:G ratio observed under moderate foliar shade, but did not reduce the R:FR ratio low enough. On their own, PG filters did not simulate any type of foliar shade. Different brands of the same filter type also had disparate effects on spectral quality. Layered CTB and ND filters improved the accuracy of moderate foliar shade simulations, and layering CTB, PG, and ND filters led to accurate simulations of deep foliar shade.

**Conclusions:**

Layering photoselective filters with contrasting effects on the spectral quality of sunlight results in more accurate simulations of foliar shade compared to when these filters are used separately. Layered filters can re-create the spectral motifs of moderate and deep foliar shade; they could be used to simulate shade scenarios found in different cropping systems. Photoselective filters offer numerous advantages over neutral density shade cloth and could be a direct replacement for researchers currently using neutral density shade cloth.

**Supplementary Information:**

The online version contains supplementary material available at 10.1186/s13007-022-00844-8.

## Background

Shade is a consistent issue for agronomic crops and horticultural plants, and there has been a considerable amount of research on the fundamental biology of shade responses, well reviewed by Casal [1] and Ballaré and Pierik [2]. Results of shade related research have also led to the modification of agronomic practices, such as alterations in plant density or spacing of row crops [3–5], or the cultivation of varieties that perform better under shade [6]. However, shade is still an issue for practitioners across many systems such as turfgrasses and landscape plants as well as in agricultural systems that use intercropping or agroforestry [7, 8].

The relative lack of improvement in tolerance towards foliar shade can be partially attributed to its complicated nature. Foliar shade, which is defined as shade due to neighboring/overhead leaves, leads to reductions in photosynthetic photon flux (PPF; photon flux between 400 and 700 nm [µmol m^−2^ s^−1^]), reductions in ultraviolet (UV) light, as well as alterations to the spectral quality of the solar radiation filtered through the foliage [9–11]. These alterations in spectral quality include distinct changes in the ratio of red to far-red (R:FR) light and in the ratio of blue to green (B:G) light that are sensed/signaled by plant photoreceptors, leading to a wide range of molecular, biochemical, and whole-plant physiological changes [12].

Phytochromes are photoreversible photoreceptors that sense red and far-red light, and isomerize upon absorption of either wavelength [11]. The red absorbing isomer (P_r_) maximally absorbs light at 660 nm and converts to the far-red absorbing isomer (P_fr_), which maximally absorbs light at 730 nm. When P_fr_ absorbs far-red light, it then photoconverts back to the P_r_ isomer [11]. Plants have been shown to respond to reductions in the ratio of the quantity of P_fr_ to the total amount of phytochrome, termed the phytochrome photoequilibrium (PPE; P_fr_/total phytochrome, or the phytochrome photostationary state) [11]. The PPE can be estimated using spectrophotometric data [13], but is influenced by weighting factors that take into account items such as phytochrome extinction coefficients and quantum yields of photoconversion [14, 15]. Weighting factors from various authors differ due to the source and quality of purified phytochrome used to develop said weighting factors [16], but the most commonly used factors from Kelly and Lagarias [17] and Sager et al. [13] lead to similar results when on a normalized scale [15]. The PPE can also be estimated using various ratios of red and far-red photon flux. Smith [11] defined the R:FR ratio calculation as the photon flux in 10 nm bands centered around 660 and 730 nm, respectively (655–665 nm/725–735 nm), but researchers have used different bandwidths including broadband calculations (600–700 nm/700–800 nm). Calculations using larger bandwidths can lead to changes in the magnitude of the R:FR ratio or potentially overestimate plant responses [9, 15].

Blue light photoreceptors include cryptochromes and phototropins [1]. Cryptochromes are blue light photoreceptors with a flavin adenine dinucleotide (FAD) chromophore that absorb maximally at ~ 450 nm [18, 19]. Green light has also been shown to be antagonistic to cryptochrome activated blue light responses. For example, the reduced cryptochrome chromophore absorbs broadly at all wavelengths of green light (500–600 nm) [19, 20], and wavelengths including 531, 540, 567, 582, and 591 nm have been shown to specifically decrease cryptochrome 2 response to blue light [18]. Phototropins contain a flavin mononucleotide (FMN) chromophore that absorbs maximally at 447 nm as well as absorbing at red wavelengths of light in its photocycle [21].

Under foliar shade, reductions in the R:FR ratio and/or the PPE occur due to red light absorption and far-red light transmission by the upper canopy, provoking specific changes in growth of the understory plants, termed shade avoidance responses (or shade avoidance symptoms). Reductions in the R:FR ratio can serve as a developmental cue due to perception and signaling phytochrome photoreceptors that have been thoroughly reviewed [1, 2, 22]. Blue (400–500 nm) and green (500–600 nm) photon flux are also altered under foliar shade due to properties of absorption, transmission, and reflection of the upper canopy leaves [1, 23, 24]. Relatively greater reductions in blue light occur under deep (forest) shade canopies [23, 24], and a relative increase in blue light is found under moderate (woodland) shade canopies [24]. Independent of blue light responses, green light has been touted to be an important source for photosynthesis in shaded plants (reviewed by Smith et al. [23]). Modifications in the B:G ratio can lead to photoreceptor-dependent shade avoidance type responses through cryptochrome photoreceptors [22, 23, 25, 26], and reductions in blue PPF, regardless of green PPF, can lead to alterations in growth and devlopment through cryptochrome and phototropins [25, 27]. Blue light triggered stomatal opening and chloroplast movement have also been shown to be inhibited or reduced by green light, with maximal action at 540 nm for stomatal opening reversal [28], and at 510, 550, and 590 nm for chloroplast movement; this is likely independent of phototropin or cryptochrome action [26]. Therefore, alterations in the B:G ratio, relatively lower amounts of blue PPF, or relatively greater amounts of green light PPF have been hypothesized to serve similar and distinct functions to phytochromes and the R:FR ratio in environmental sensing and acclimation to foliar shade [23].

Further complicating whole-plant responses to foliar shade, ultraviolet light has been shown to suppress shade avoidance responses [29, 30]. Hayes et al. [29] showed that treatment of *Arabidopsis* plants with UV-B (280–315 nm; 400 mW m^−2^ [~ 1 µmol m^−2^ s^−1^]) inhibited the shade avoidance responses brought upon treatment with a very low R:FR ratio of 0.05. Perception and signaling of UV-B light occurs through the photoreceptor UV resistance locus 8 (UVR8), which has been shown to mediate the inhibition of shade avoidance response specifically under UV-B light [29, 30]. Recently, Rai et al. [31] showed that UVR8 also absorbs and mediates responses to short-wave UV-A photons (315–350 nm), while cryptochromes mediate responses to long-wave UV-A photons (350–399 nm). Additionally, UVR8 absorbs more short-wave UV-A relative to UV-B [31] suggesting a potential larger role of UV-A light in photomorphogenesis under foliar shade.

Altogether, UV, blue, green, red, far-red light, and the spectral ratios described have additive and independent effects on plants under foliar shade. The R:FR and B:G ratios have been shown to have overlapping effects on shade avoidance responses, and separate effects such as green light’s role in the reversal of blue light responses, like stomatal opening [23]. Therefore, because these signals can lead to different responses, it is important that they are all examined in whole-plant foliar shade experiments.

It can be difficult for researchers to examine whole-plant tolerance to altered spectral quality without also dealing with other stresses, such as water-deficit, and simulations are therefore needed to remove these confounding effects. Many researchers use neutral density black shade cloth (shade cloth, black shade cloth, etc.) to apply shade treatments in the field or in greenhouses, but this only reduces PPF and does not alter spectral quality [32, 33]. Plants respond differently to altered spectral quality compared to reductions in PPF alone [34–36], and as discussed previously, alterations in the R:FR ratio, the B:G ratio, and UV photon flux specifically regulate plant growth and development. Using neutral density black shade cloth may lead to misinterpretation of a plants’ tolerance to foliar shade, and expression or post-translational regulation of foliar shade specific genes may not occur under neutral density black shade cloth treatment, limiting the ability to make genetic improvements for tolerance to foliar shade.

**Fig. 1:**
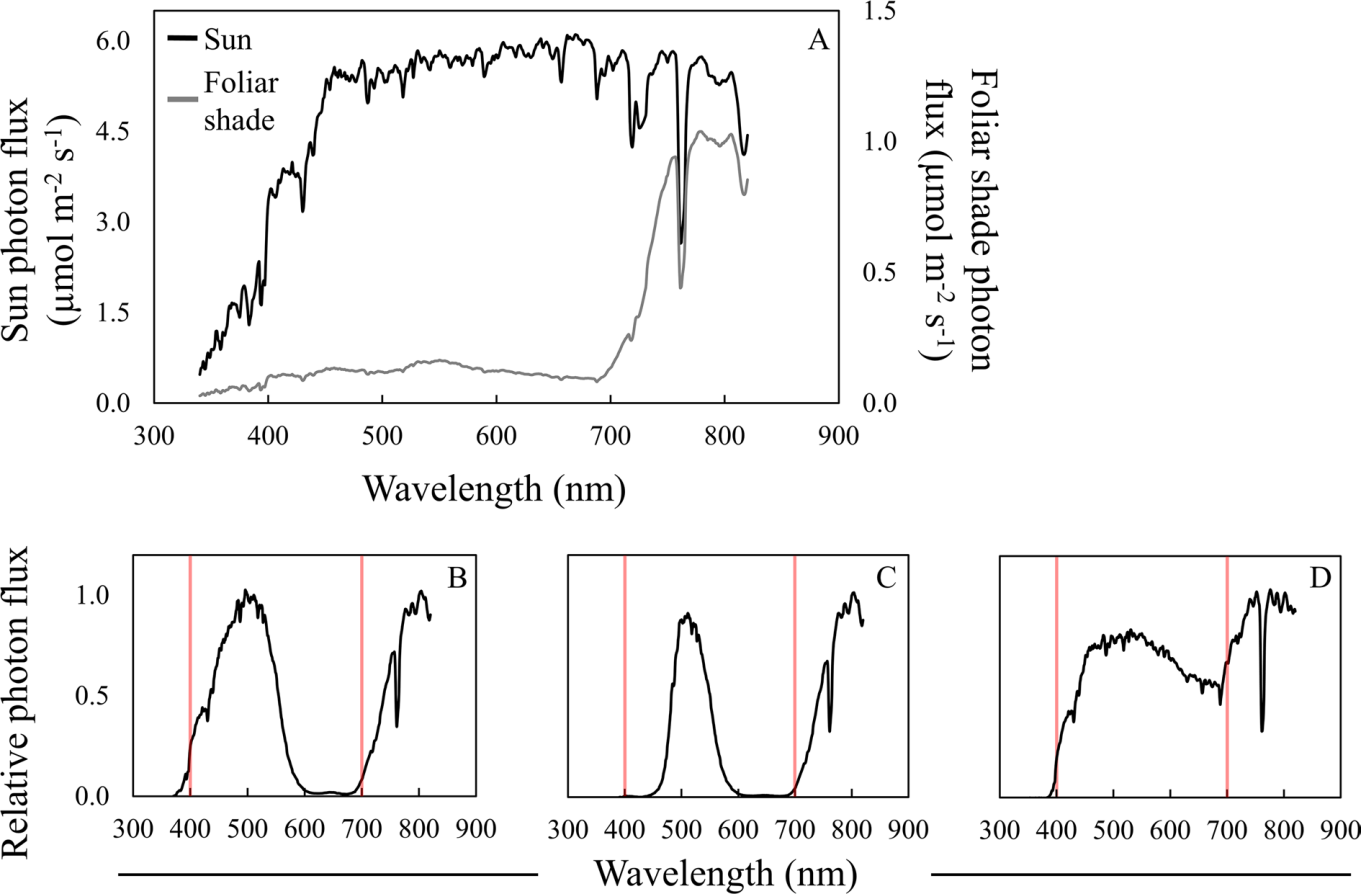
Photoselective filters used in previous research do not simulate foliar shade well. **A** A generalized spectral photon distribution (SPD) of sun and foliar shade (left and right y-axes are scaled differently to represent differences in light intensity while maintaining visible differences in spectral quality). **B** SPD of LEE Filters Peacock blue filter used by Runkle and Heins [42] (figure modified from Petrella and Watkins [48]). **C** SPD of LEE Filters Dark green filter used by [35] (figure modified from [48]). **D** SPD of a Mitsubishi blue polyethylene filter used by Petrella and Watkins [40] and Studzinska et al. [44] (figure modified from Petrella and Watkins [40]). Red bars indicate 400 and 700 nm, respectively, designating photosynthetically active radiation (PAR) between the red bars

Materials that selectively filter light exist and have been used to simulate foliar shade or examine wavelength specific responses in plant science research [37–44]. Photoselective filters (i.e. photoselective gels or interference filters) are thin polyester plastic sheets containing dyes that selectively filter wavelengths of light [45]. Filters used in more recent research, such as Peacock blue and Dark green, lead to alterations in spectral quality that are more extreme than what occurs under foliar shade (Table 1; Fig. 1); these filters almost completely remove red light (600–700 nm) to achieve a strong reduction in the R:FR ratio (Fig. 1). Hurdzan and Klein [38] aimed to improve foliar shade simulations through layering a Medium amber Cinemoid filter with a Slate blue Cinemoid filter, and while these authors showed that specific spectral ratios like the R:FR ratio simulated deciduous shade to some degree, they failed to show if this system accurately simulated the entire deciduous shade spectral photon distribution (SPD). Simply using filters that have been used in previous research, whether or not they were used in shade research specifically, may not be the best choice for those looking to simulate foliar shade more accurately.

**Table 1:** Attributes of photoselective filters used in previous research

Filter(s) used	R:FR^a^	PPE^b^	B:G^c^	Additional comments	Author(s)
Cinemoid Medium amber (No. 4) + Cinemoid Slate blue (No. 61)	0.75	–	–	Data were collected under sunlight in a greenhouse near solar noon	Hurdzan and Klein [38]
Cinemoid Primary green (No. 39)	–	0.03	–	Data were collected in a growth chamber with fluorescent lamps	Hilton et al. [46]
Green plastic film	0.69–0.83	–	–	Data were collected under sunlight in the field, no mention of time of day	Skálová and Krahulec [43]
LEE Filters, Dark green (No. 124)	0.04	0.44	–	Data were collected in a growth chamber with metal halide lamps	Gautier et al. [35]
LEE Filters, Peacock blue (No. 115)	0.04	0.39	–	Data were collected under sunlight in a greenhouse, no mention of time of day; Under high pressure sodium lamps, R:FR = 0.12, PPE = 0.62	Runkle and Heins [42]
LEE Filters, Soft green (No. 322)	0.10	–	–	Data were collected under sunlight in a greenhouse, no mention of time of day	Gautier et al. [47]
LEE Filters Pale green (No. 138)	0.70	–	–	Data were collected under sunlight in a greenhouse, no mention of time of day	Griffith and Sultan [37]
Rosco, Roscolux Surprise pink (No. 51)	0.58	–	–	Data were collected in a growth chamber with fluorescent and incandescent lamps	Linkosalo and Lechowicz [39]
Mitsubishi, blue polyethylene	0.66—0.70	0.67	–	Data were collected under sunlight in a greenhouse near solar noon	Petrella and Watkins [40] Studzinska et al. [44]

^a^Red to far-red light ratio

^b^Photosynthetic photoequilibria

^c^Blue to green light ratio

We previously evaluated 85 blue, green, or neutral density (ND) filters from two companies, LEE Filters and Rosco, and observed that while many filters are well suited to simulate specific R:FR ratios, only a select number of filters are useful for simulating an entire SPD including color temperature blue (CTB) and ND filters [48, 49]. However, while the spectral properties of other filters may not be well suited to simulate foliar shade on their own, some filters, including plus green (PG) filters, may improve foliar shade simulations when layered with contrasting filters. Additionally, because shade simulations may take place in greenhouses, common supplemental lighting sources such as high-pressure sodium (HPS) or metal halide (MH) lamps may further affect the spectral quality underneath these filters due to differences in their spectral output [50]. Therefore, our overall objective was to examine if single-, double-, and triple-layered photoselective filters consisting of CTB, PG, or ND filters from LEE Filters and Rosco improved the accuracy of simulating spectral quality of foliar shade under both natural and electric lighting.

## Materials and methods

### Description of the photoselective filters evaluated

**Fig. 2:**
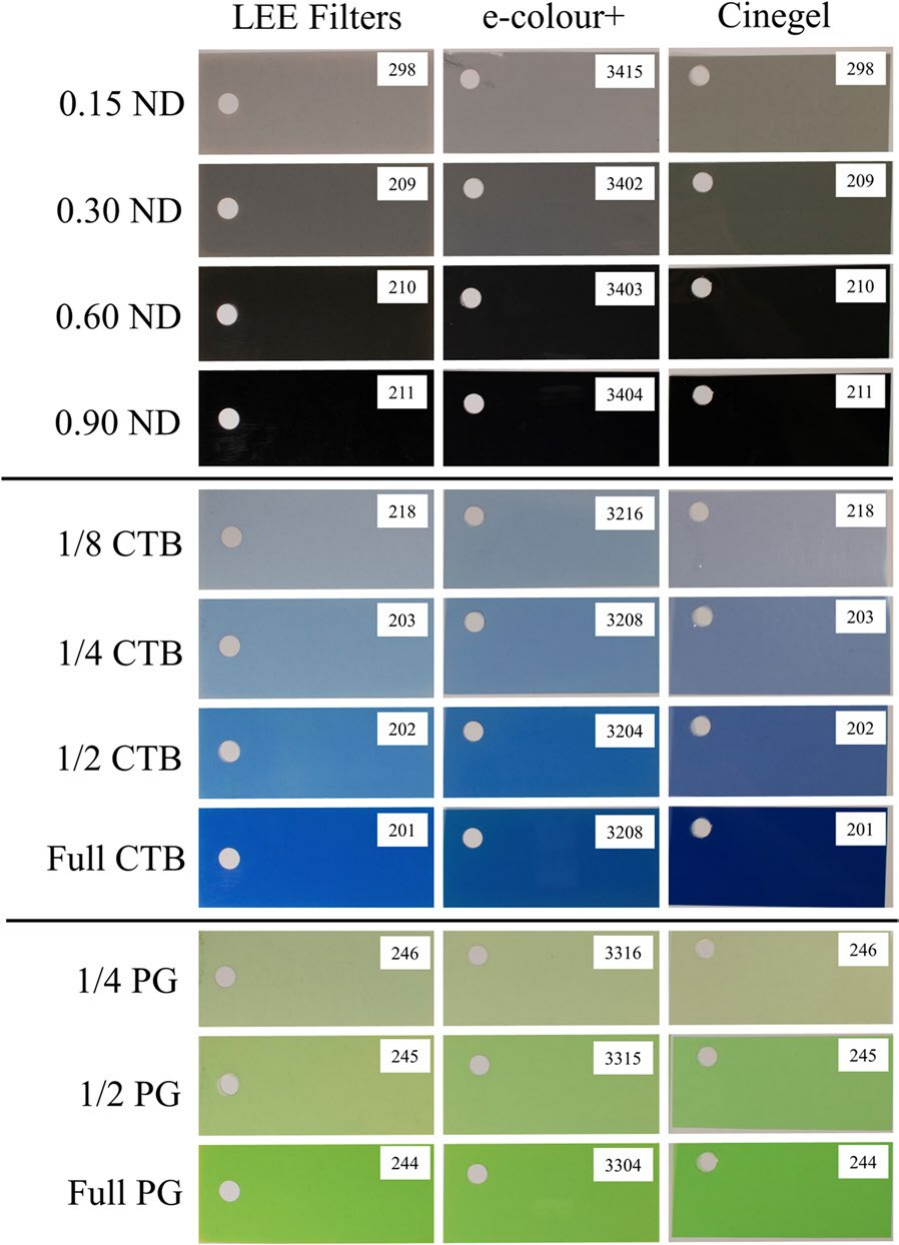
Digital images of the photoselective filters from LEE Filters, Rosco e-colour + , and Rosco Cinegel used in this research. Multiple strengths of each filter were evaluated including; 0.15–0.90 neutral density (ND), 1/8—full strength color temperature blue (CTB), and 1/4—full strength plus green (PG). Model numbers of each filter are inset in the top-right corner of each filter picture

**Fig. 3:**
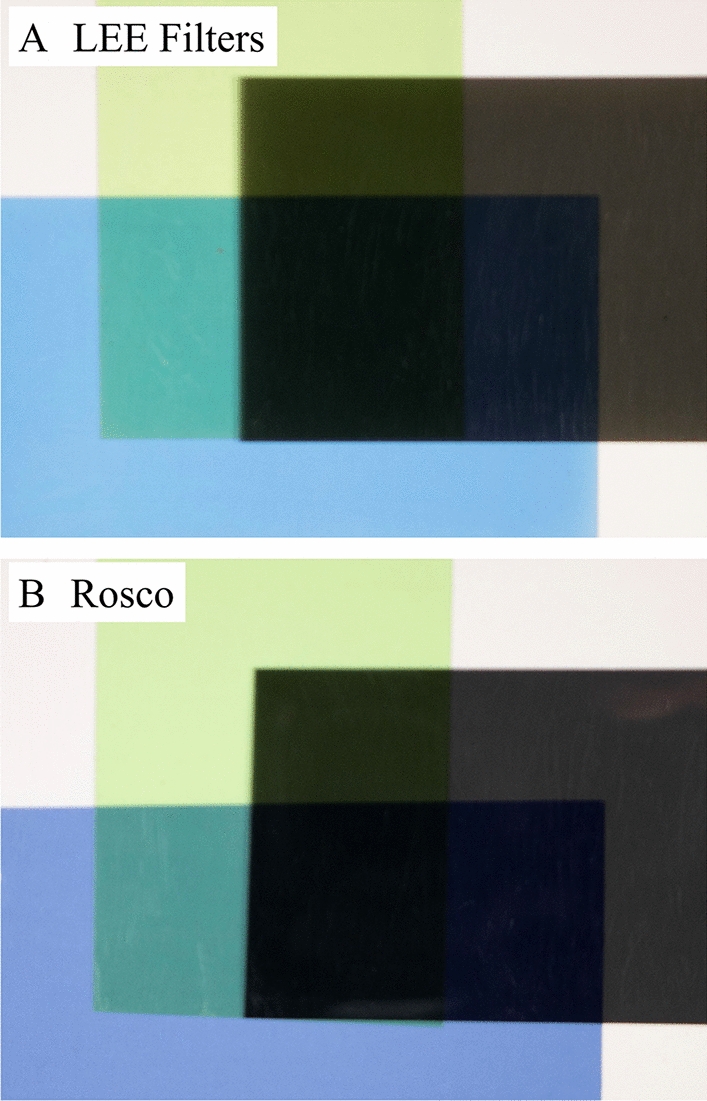
Digital images of (**A**) layered LEE Filters 1/2 color temperature blue (CTB) on the bottom, LEE Filters 1/2 plus green (PG) in the middle, and LEE Filters 0.60 neutral density (ND) filter on top. **B** Layered Rosco Cinegel 1/2 CTB on the bottom, Rosco e-colour + 1/2 PG in the middle, and Rosco e-colour + 0.60 ND filter on top

Filters were chosen based on preliminary results that indicated which filters may improve the accuracy of foliar shade simulations when layered [48, 49]. For this study we examined ND, CTB, and PG filters only. For each type of filter, we acquired data from three brands: (1) LEE Filters (Hampshire, UK), (2) Rosco Cinegel (Stamford, CT, USA), and (3) Rosco e-colour + (Stamford, CT, USA), in which the same filters from different brands were mostly indistinguishable to our eyes (Fig. 2). These filters are all available in multiple strengths, ranging from weak to strong effects on spectral quality, and we therefore evaluated multiple strengths of each filter (Fig. 2). In each case, we evaluated the alteration in spectral quality under a single layer of each filter separately, and when the filters were layered. For double-layered filters, ND filters were on top, and for triple-layered filters, CTB filters on the bottom, PG in the middle, and ND on top (Fig. 3).

### Photoselective filter spectral data collection

Spectral data were collected under photoselective filters in unobstructed, natural sunlight during sunny days in 2020 at the University of Minnesota Turfgrass Research, Outreach, and Education center. Data were collected on 27 May, 30 May, and 12 June 2020 and each day was treated as a replicate for statistical analyses. Data were also collected in greenhouses at the Minnesota Agricultural Experiment Station Plant Growth Facility on 12 June, 13 June, and 14 June 2020 at 22:00 h (when supplemental lighting was the sole light source) with either 400 W HPS (LU400/H/ECO, General Electric, Boston MA, USA) or 400 W quartz MH (MVR400/U, General Electric, Boston MA, USA) high-intensity discharge lamps to determine the maximum potential effects of these common sources of supplemental lighting on the spectral effects of the photoselective filters.

A cosine corrected spectroradiometer (Apogee Instruments SS-110 [340–820 nm], Logan UT, USA) was placed on a box and was leveled ~ 15.24 cm from the surface to collect spectral data. Another box with a ~ 3.0 × 3.0 cm hole was placed over the spectroradiometer. The sensor was positioned under the hole such that it was even with surface of the outside of the box. Sections of photoselective filter (7.62 × 3.81 cm) were then placed over the 3.0 × 3.0 cm hole for the sensor to only be exposed to sunlight filtered through the filter (Additional file 1: Fig. S1). An automatic integration time was used while taking data, and data were acquired using an average of three scans. Spectral data for each filter were acquired in a random order on each day.

### Proof-of-concept: Foliar shade spectral data collection

Spectral data were acquired under foliar shade and in an area of unobstructed full sun (Additional file 1: Fig. S2) at the University of Minnesota Turfgrass Research, Outreach, and Education center (44°59′42.31″N, 93°11′10.25″W) and the University of Minnesota St. Paul campus (44°59′10.32″N, 93°11′04.05″W) to determine the effectiveness of the photoselective filters to simulate these spectral data.

Data were acquired in a five row by four column (north–south) grove of sugar maples (*Acer saccharum*) that were ~ 9–12 m in height and on 6 m spacings. Data were acquired on the south end (Maple grove-southern row) of the grove (between rows one and two), and data were taken on the north end (Maple grove-northern row) of the grove (between rows four and five). Data were acquired within a mature grove of northern red oaks (Oak grove, *Quercus rubra*) that were ~ 30–37 m in height and of a mixed spacing. Data were acquired on the north edge of a small mixed-species forest (Northern forest edge) that had unevenly spaced young Ohio buckeye trees (*Aesculus glabra*) to the north (9–12 m in height). Data were taken on the south side of the same small forest (Southern forest edge) that also had a single, mature, Norway maple (*Acer platanoides*) just to the south (21–24 m in height), and data were acquired between the Norway maple and the southern forest edge. Data were taken within the small mixed-species forest (Within a forest) in approximately the midpoint between Southern and Northern forest edge sites. In 2018, data were collected on 28 May, 13 June, 2 July, and 6 July for sites 1–4 and 6, and on 30 May, 31 May, 12 June, and 11 Aug. 2020 for site 5. Data were acquired between 13:00 and 14:00 h for all sites on clear sky or mostly sunny days only.

We also collected data under three agronomic crops: wheat (*Triticum aestivum*), barley (*Hordeum vulgare*), and canola (*Brassica napus*) in 2018 (Additional file 1: Fig. S3). Wheat data were collected within a field that was seeded on 14 May 2018 in St. Paul MN, and spectral data were collected on 2 and 6 July 2018. Twelve independent scans under wheat were collected in between rows, at least four rows in from plot edges, and were collected in random locations. Data for both barley and canola were collected under plants seeded in six rows on 4 March 2018 in a greenhouse in St. Paul MN. Six independent scans were taken between rows 3 and 4 for both canola and barley on 23 April 2018 only.

To collect data under foliar shade, a spectroradiometer was placed directly on the turfgrass/soil surface, and was leveled to only measure vertical flux. An automatic integration time was used while taking data, and data were acquired using an average of three scans in Apogee SpectroVision software.

### Data analysis

For the spectral data collected, we evaluated the overall SPD, and also calculated specific spectral ratios and reductions in PPF as follows: (1) R:FR ratio = 655–665 nm/725–735 nm [11]; (2) the PPE [derived within SpectroVision software; [13]]; (3) B:G ratio = 420–490 nm/500–570 nm [25]; (4) the relative quantity of blue (400–499 nm), green (500–599 nm), and red (600–700 nm) photon flux to the total PPF; (5) reduction in PPF relative to full sun; and (6) long-wave UV-A photon flux (340–399 nm) as an indicator of potential total UV light transmittance. All SPD data were normalized to 800 nm. Data collected under foliar shade and agronomic crops in the field were used as a reference to evaluate the accuracy of the photoselective filters as a proof-of-concept, and we did not make any statistical comparisons between these foliar shade data. These data are presented as the mean ± the standard deviation. Data for spectral ratios and reductions in the PPF collected under photoselective filters were subjected to ANOVA using a mixed model where filter was treated as a fixed effect, replicate (date of data acquisition) was treated as a random effect, and the interaction between filter and replicate was treated as a random effect. Means were compared using Fisher’s protected LSD (*P* = 0.05). All data were analyzed using JMP^®^ version 14.0 (SAS Institute Inc., Cary, NC, USA).

For this research we chose to evaluate the narrowband R:FR ratio based on the maximal absorption of P_r_ (660 nm) and P_fr_ (730 nm) isomers, the photon flux in 10 nm bands centered around 660 and 730 nm, respectively (655–665 nm/725–735 nm). As stated by Smith [11], many researchers characterize the R:FR ratio using different bandwidths, including broadband calculations (600–700 nm/700–800 nm), leading to different ratios that may not be able to be compared. We chose to analyze our data using the narrowband R:FR ratio (655–665 nm/725–735 nm) due to this method being a classical approach that more researchers, across multiple disciplines, may be familiar with, and because our data show that this ratio is highly correlated with broadband calculations (600–700 nm/700–800 nm).

We examined alterations in the B:G ratio under foliar shade and photoselective filters using bandwidths (420–490 nm/500–570 nm) previously shown to be effective at predicting *Arabidopsis* hypocotyl length [25]. While green light has been shown to be antagonistic to blue light responses, to our knowledge few have examined specific B:G ratios. This green light bandwidth, in particular, includes peak wavelengths shown to result in known blue light photoreceptor dependent and independent action [18, 20, 26, 28]. Because of the lack of a highly studied narrowband B:G ratio known to be highly correlated with plant response, we also examined the correlation between the relatively narrowband bandwidths described by Sellaro, et al. [25], used here, and broadband calculations of the B:G ratio (400–500 nm/500–600 nm). For both narrow- and broadband calculations of the R:FR and B:G ratios we examined correlations and rank change, Pearson’s correlations using linear regression analyses and Spearman’s rank analysis. All data were analyzed using JMP^®^ version 14.0.

## Results and discussion

### Narrowband and broadband spectral ratio correlations and rank analyses

Different bandwidths can be used to calculate the R:FR and B:G ratios. To examine alterations in spectral quality under foliar shade and photoselective filters we calculated these ratios in relatively narrow bands, R:FR = 655–665 nm/725–735 nm and B:G = 420–470 nm/500–570 nm, based on previously defined calculations [11, 25]. To determine if broadband calculations would lead to different conclusions on photoselective filter simulations, we examined correlations and alterations in rank between narrow- and broadband R:FR and B:G spectral ratios (Additional file 1: Figs. S4–S7).

For data collected under foliar shade in the field, the narrow- and broadband R:FR ratios were highly correlated (r^2^ = 0.994, *p* < 0.0001) and there were no significant changes in foliar shade site rank when either calculation was used (Spearman’s ρ = 0.9953, *p* < 0.000). For data collected under photoselective filters or layered photoselective filters the narrow- and broadband R:FR ratios were highly correlated (r^2^ = 0.984, *p* < 0.0001) and there were no significant changes in filter ranking when either calculation was used (Spearman’s ρ = 0.9920, *p* < 0.000). Lastly, for data collected under photoselective filters in a greenhouse with supplemental lighting (HPS and MH sources) the narrow- and broadband R:FR ratios were highly correlated (r^2^ = 0.9950, *p* < 0.0001) and there were no significant changes in filter ranking when either calculation was used (Spearman’s ρ = 0.9924, *p* < 0.000). For all R:FR bandwidth correlations, data fell within a 95% confidence interval for value prediction (Additional file 1: Figs. S4–S7).

Under foliar shade, the narrow- and broadband B:G ratios were highly correlated (r^2^ = 0.982, *p* < 0.0001) and there were no significant changes in foliar shade site rank when either calculation was used (Spearman’s ρ = 0.9676, *p* < 0.000). Removing full sun data from these analyses improved the correlation (r^2^ = 0.994, *p* < 0.0001) and decreased the probability of rank change (Spearman’s ρ = 0.9880, *p* < 0.000) (Additional file 1: Fig. S5). The broadband calculation resulted in relative reductions in the magnitude of B:G ratio in full sun, while under all foliar shade sites the broadband calculation either increased or led to no significant change compared to the narrowband calculation (Additional file 1: Fig. S5). For data collected under photoselective filters or layered photoselective filters the narrow- and broadband B:G ratios were highly correlated (r^2^ = 0.983, *p* < 0.0001) and there were no significant changes in filter ranking when either calculation was used (Spearman’s ρ = 0.9920, *p* < 0.000). Only data for Rosco e-colour + 1/2 and Full CTB and LEE Filters Full CTB were outside of the 95% confidence interval for value prediction (Additional file 1: Fig. S6). For data collected under photoselective filters in a greenhouse with supplemental lighting, the narrowband and broadband B:G ratios were well correlated (r^2^ = 0.961, *p* < 0.0001) and there were no significant changes in filter ranking when either calculation was used (Spearman’s ρ = 0.9852, *p* < 0.000). Only data for Rosco e-colour + and LEE Filters Full CTB were outside of the 95% confidence interval for value prediction (Additional file 1: Fig. S7).

The bandwidths used to calculate the R:FR and B:G ratio may alter the magnitude of these spectral ratios, but our data show that even under photoselective filters with or without MH/HPS supplemental lighting, both narrow- and broadband calculations would lead to the same overall conclusions drawn about photoselective filters here. On the other hand, light emitting diode (LED) based supplemental lighting could lead to discrepancies between different bandwidth calculations due to their discrete, non-continuous, spectral distribution, potentially worsened due to differences in LED standards between manufacturers [51].

### Foliar shade SPDs and spectral ratios

**Fig. 4:**
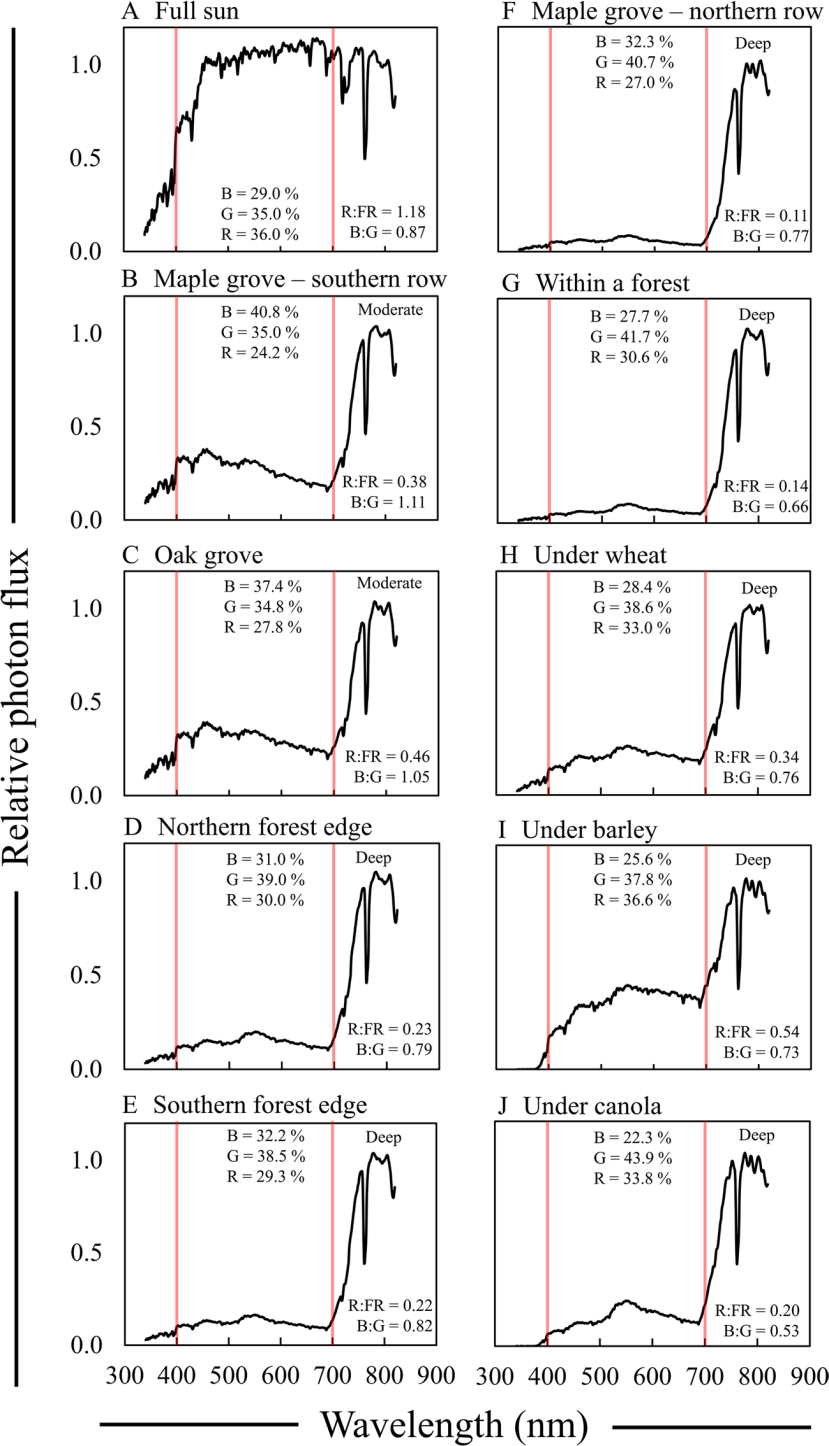
Relative spectral photon distribution (SPD) data acquired under different foliar shade sites representing moderate and deep spectral motifs in St. Paul MN during 2018 and 2020. **A** Full sun, **B** Maple grove-southern row, **C** Oak grove, **D** Northern forest edge, **E** Southern forest edge, **F** Maple grove-northern row, **G** Within a forest, **H** Under a wheat canopy, **I** Under a barely canopy in a greenhouse, and **J** Under a canola canopy in a greenhouse. Data were normalized to the photon flux at 800 nm and are presented as the average of relative SPDs acquired on 28 May, 13 June, 2 July, and 6 July for 2018 (**A**–**E**, **G**), 30 May, 31 May, 12 June, and 11 Aug. 2020 (**F**), 2 and 6 July 2018 (**H**), and 23 April 2018 (**I**, **J**). The average relative PPF of blue (B), green (G), and red (R) light are inset in each panel (see Additional file 2: Table S1 for sample statistics); the average R:FR (655–665 nm/725–735 nm) and B:G (420–490 nm/500–570 nm) ratios are inset in each panel. Data were acquired between 13:00 and 14:00 h on clear sky or mostly sunny days only. Red bars indicate 400 and 700 nm, respectively, designating photosynthetically active radiation (PAR) between the red bars

Significant reductions in the R:FR ratio and changes in the relative amount of blue light were observed between foliar shade sites and under the agronomic crops evaluated relative to full sun (Fig. 4; Table 2). The range in R:FR ratios observed across all the sites we evaluated was similar with previous research examining spectral changes under various species of trees and crops [9, 36, 52–56].

**Table 2:** Average data collected under either full sun or foliar shade

Site	R:FR^a^	PPE^b^	B:G^c^	PPF reduction^d^	UV-A PF^e^
				%	µmol m^−2^ s^−1^
Full sun^f^	1.18 ± 0.03^g^	0.72 ± 0.00	0.87 ± 0.01	–	85.18 ± 7.98
Maple grove-southern row	0.38 ± 0.07	0.57 ± 0.02	1.11 ± 0.06	94.0 ± 1.4	12.74 ± 1.79
Oak grove	0.46 ± 0.06	0.60 ± 0.01	1.05 ± 0.07	95.0 ± 0.8	9.02 ± 1.17
Northern forest edge	0.23 ± 0.06	0.48 ± 0.04	0.79 ± 0.05	97.8 ± 0.5	3.33 ± 0.28
Southern forest edge	0.22 ± 0.03	0.46 ± 0.03	0.82 ± 0.03	98.0 ± 0.8	2.78 ± 0.14
Maple grove-northern row	0.11 ± 0.03	0.34 ± 0.04	0.77 ± 0.03	99.0 ± 0.0	1.58 ± 0.24
Within a forest	0.14 ± 0.02	0.37 ± 0.02	0.66 ± 0.04	99.0 ± 0.0	0.90 ± 0.15
Under wheat	0.34 ± 0.08	0.53 ± 0.06	0.76 ± 0.07	93.3 ± 0.3	6.40 ± 0.99
Under barley	0.54 ± 0.09	0.62 ± 0.03	0.73 ± 0.03	78.7 ± 5.8	1.79 ± 0.55
Under canola	0.20 ± 0.08	0.46 ± 0.06	0.53 ± 0.07	93.6 ± 2.5	0.45 ± 0.20

^a^R:FR = 655–665/725–735

^b^PPE = Phytochrome photoequilibria

^c^B:G = 420–490/500–570

^d^PPF reduction = Percent reduction in PPF relative to full sun. Full sun PPF was on average 1617 ± 209 µmol m^−2^ s^−1^

^e^UV-A PF = Photon flux between 340 and 399 nm

^f^Site description

^g^Mean ± standard deviation; Data are presented as the average from the following dates for each site: 28 May, 13 June, 2 July, and 6 July for 2018 (full sun, sites 1–4, and site 6), 30 May, 31 May, 12 June, and 11 Aug. 2020 (site 5), 2 and 6 July 2018 (wheat), and 23 April 2018 (barley and canola). Data were acquired between 13:00 and 14:00 h on clear sky or mostly sunny days only

Deep shaded areas, such as the Maple grove-northern row and Within a forest, along with agronomic crops had a greater amount of green light (500–600 nm) relative to the other wavelengths (Fig. 4D–J; Table 2) [7, 8]. While green light on its own and its proportion to blue light are known to effect plant growth and development, to our knowledge few published papers have presented extracted B:G ratio data from the overall SPD. However, Sellaro et al. [25] showed that under dallisgrass (*Paspalum dilatatum*), the B:G ratio could be as low as 0.30. The relatively greater amount of green light compared to blue light could exacerbate shade avoidance responses of understory plants through antagonizing cryptochrome signaling, as well as reversing blue-light induced stomatal opening, potentially decreasing gas exchange [18, 28].

In moderately shaded sites, the Maple grove-northern row and the Oak grove, there was a prominent increase in the B:G ratio and the percentage of blue light compared to full sun (Fig. 4B, C; Table 2). McKee [57] indicated that shade underneath trees with a higher canopy is more enriched in blue photons, akin to data collected under the Oak grove. Similar observations have been shown to occur higher in forest canopies [52, 58] where more light diffuses in, and a relative increase in blue light has been shown to occur in woodland canopies as well [24]. A relative increase in the amount of blue light compared to green light may lead to some repression of cryptochrome-mediated shade avoidance responses of the understory plants, and potentially result in greater carbon fixation due to increased stomatal opening [59]. Relatively small increases in blue PPF (less than 1.0 µmol m^−2^ s^−1^) have also been shown to improve growth under an overall low PPF, mediated by phototropins [27].

The quantity of UV-A (340–399 nm) photons drastically declined in all shade sites compared to full sun with reductions ranging from a ~ 7–190 fold decrease (Table 2). While the spectroradiometer used in these experiments did not quantify UV-B photon flux, we hypothesize, based on previous research that any UV-B photon flux would decrease under these foliar shade sites by similar magnitudes observed with UV-A photon flux [60]. Photoselective filters or filter combinations that simulate changes in UV light along with changes in PPF and far-red light may be best suited for whole-plant shade simulations, as UV light has been shown to inhibit shade avoidance responses at low a photon fluence [29].

### Photoselective filter SPDs and spectral ratios

**Fig. 5:**
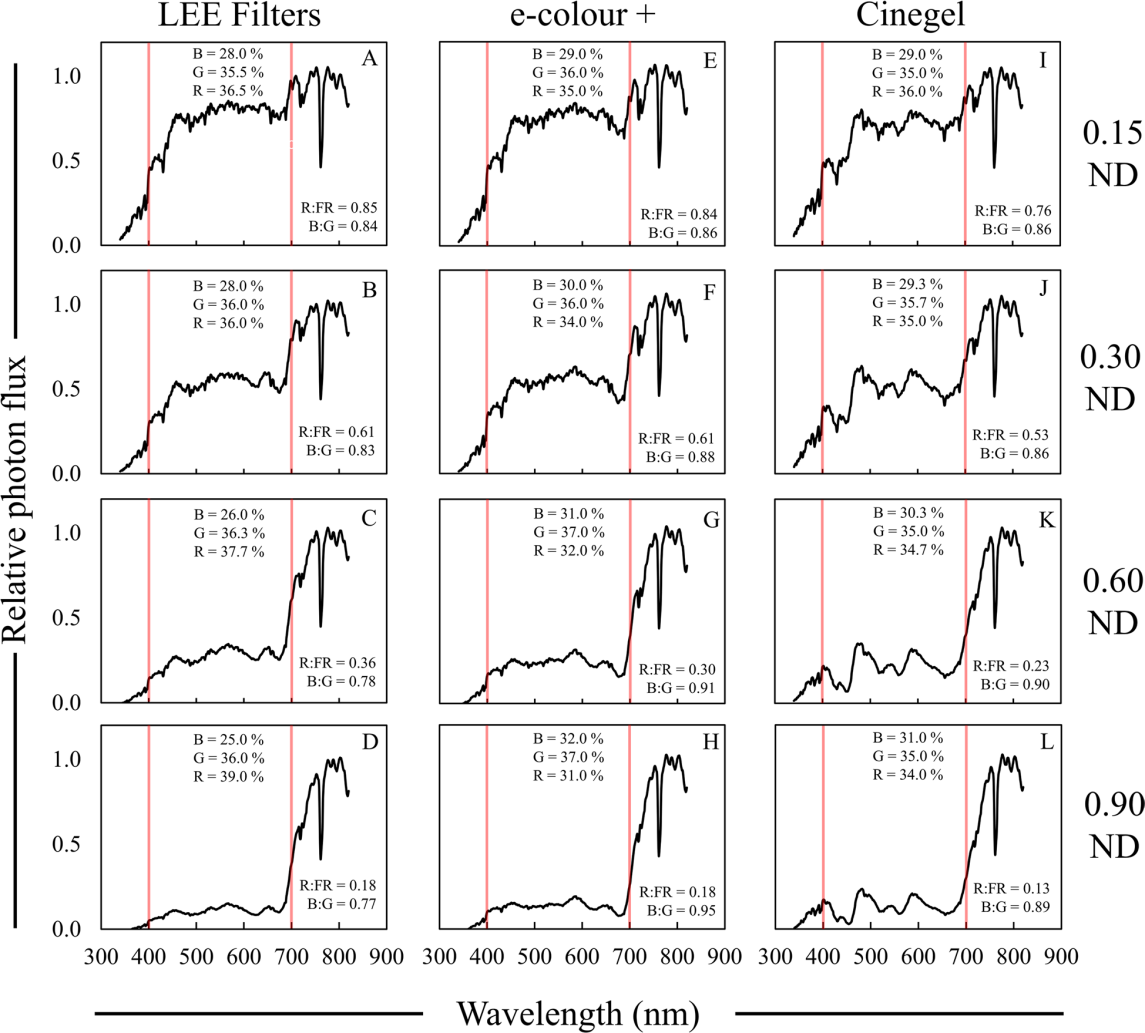
Relative spectral photon distribution (SPD) data acquired under 0.15–0.90 strength neutral density (ND) filters from LEE Filters (**A**–**D**), Rosco e-colour + (**E**–**H**), and Rosco Cinegel (**I**–**L**). Data were normalized to the photon flux at 800 nm and are presented as the average of relative SPDs acquired on 27 May, 30 May, and 12 June 2020 on clear sky or mostly sunny days between 13:00–14:00 h. The average relative PPF of blue (B), green (G), and red (R) light are inset in each panel (see Additional file 2: Table S2 for sample statistics); the average R:FR (655–665 nm/725–735 nm) and B:G (420–490 nm/500–570 nm) ratios are inset in each panel. Data collected under full sun were on average; R:FR = 1.15, B:G = 0.87, % Blue = 29%, % Green = 35%, and % Red = 36%. Red bars indicate 400 and 700 nm, respectively, designating photosynthetically (PAR) active radiation between the red bars

For all evaluated ND filters there was a non-linear reduction in the R:FR ratio with increased filter strength. This feature was previously noted by Jackman [45] to occur when using ND filters for photography, and our data shows that strength of the filter (i.e. full, 0.15, 0.60, 1/2, 1/4, etc.) does not necessarily indicate the strength of the modification to spectral quality. LEE and Rosco e-colour + ND filters were not significantly different (except for 0.60 ND filters) for the R:FR ratio, but Rosco Cinegel ND filters led to significantly lower R:FR ratios compared to the other two brands (Table 3), more than likely due to the non-neutral PPF reductions from Cinegel ND filters (Fig. 5I–L).

**Table 3:** Average data collected neutral density (ND) photoselective filters

Filter	Brand	R:FR^a^	PPE^b^	B:G^c^	PPF reduction^d^	UV-A PF^e^
					%	µmol m^−2^ s^−1^
0.15 ND	LEE	0.85 A^f^	0.69 A	0.84 F	29.7 H	50.4 BC
	e-colour +	0.84 A	0.68 B	0.86 E	36.0 G	45.5 C
	Cinegel	0.76 B	0.68 B	0.86 E	36.0 G	66.3 A
0.30 ND	LEE	0.61 C	0.64 C	0.83 G	51.7 F	27.3 DE
	e-colour +	0.61 C	0.64 C	0.88 D	53.0 EF	32.3 D
	Cinegel	0.53 D	0.64 C	0.86 E	54.0 E	54.1 B
0.60 ND	LEE	0.36 E	0.56 D	0.78 H	76.0 D	10.1 FG
	e-colour +	0.30 F	0.53 E	0.91 B	79.7 C	12.5 F
	Cinegel	0.23 G	0.53 E	0.90 BC	79.7 C	32.6 D
0.90 ND	LEE	0.18 GH	0.42 H	0.77 I	90.0 A	2.5 H
	e-colour +	0.18 GH	0.43 G	0.95 A	88.3 AB	6.1 GH
	Cinegel	0.13 H	0.45 F	0.89 CD	88.0 B	24.4 E

^a^R:FR = 655–665/725–735

^b^PPE = Phytochrome photoequilibria

^c^B:G = 420–490/500–570

^d^PPF reduction = Percent reduction in PPF relative to full sun

^e^UV-A PF = Photon flux between 340 and 399 nm

^f^Data are presented as averages acquired on three different clear sky or mostly sunny days between 13:00 and 14:00 h: 27 May, 30 May, and 12 June 2020. Means are only compared within column and were separated with Fisher’s LSD. Means followed by a common letter are not significantly different (*P* = 0.05). Data collected under full sun were on average; R:FR = 1.15, PPE = 0.72, B:G = 0.87, PPF = 1719 µmol m^−2^ s^−1^, UV-A PF = 86 µmol m^−2^ s^−^

The effects of ND filters on spectral quality can be easily confused with ND black shade cloth, which is commonly used to reduce PPF for shade experiments. Compared to ND filters, which lead to neutral reductions in photosynthetically active radiation (PAR; the wavelength region of solar radiation between 400 and 700 nm), ND black shade cloths lead to neutral reductions in all wavelengths of solar radiation, and do not alter spectral ratios like the R:FR or B:G ratios. Arthurs et al. [32] showed that black shade cloths did not alter the R:FR ratio beyond natural levels in an Apopka FL greenhouse, and Kotilainen et al. [33] showed that multiple brands of black shade cloth also did not alter the R:FR ratio beyond natural levels or lead to alterations in the overall SPD compared to full sun in Raleigh NC. Altogether, ND black shade cloths never alter spectral quality similar to that of foliar shade. Colored shade cloths are also available, but these have been shown to have limited effects on spectral quality [32, 33].

While called “neutral density”, the ND filters still did not reduce all wavelengths of PAR equally. This was most apparent with LEE Filters 0.30 ND and, in particular, 0.90 ND filters which led to a greater decrease in the relative quantity blue PPF (400–500 nm) compared to the other filters (Fig. 5). Along the same lines of neutrality, the blue light selective nature of the LEE Filters ND filters could be seen in lower B:G ratios compared to both Rosco filters (Table 3). On the other hand, the Rosco e-colour + ND filters were significantly less selective for blue light (Fig. 5; Table 3).

The quantity of UV-A light transmitted through the different ND filters was also variable from brand-to-brand as the strength of the filter increased. Rosco Cinegel filters transmitted a greater amount of UV-A compared to both LEE and Rosco e-colour + filters, no matter the filter strength, while there were no significant differences in UV-A transmission between LEE Filters and Rosco e-colour + (Table 3). Both 0.60 and 0.90 ND filters resulted in a UV-A photon flux that was similar to what was observed under moderate and deep foliar shade, respectively.

Neutral density filters alone did not provide a full simulation of foliar shade SPDs. This was most apparent for alterations to blue and green PPF, largely due to their overall PAR neutrality. Neutral changes in wavelengths of PAR did not occur under any foliar shade scenario in St. Paul MN. For example, we observed double the amount of green light compared to blue light under canola, a 14% difference between blue and green light within a forest, and ~ 8% more green photons relative to blue in other deeply shaded sites (Fig. 4). For moderate foliar shade sites, we observed a 2.5–6% increase in the amount of blue photons compared to green (Fig. 4).

Small increases in the relative amount of blue light may enhance growth under foliar shade through phototropin action, especially when the total PPF is low [27]. While larger differences between blue and green PPF may be needed (~ 1:2) to interfere with cryptochrome-mediated shade avoidance symptoms [18], previous research has indicated that relatively small differences in blue and green PPF may lead to photoreceptor-independent responses. Between 1 and 10 µmol m^−2^ s^−1^ of blue photons have been shown to effectively open stomata within 60 min in various species of ferns, and only 10 µmol m^−2^ s^−1^ were needed for significant increases in stomatal conductance [61]. In *Vivica faba*, equal proportions of blue and green light led to a 50% reduction in stomatal opening, a ratio of 0.50 led to approximately a 100% reduction, and an interpolated ratio of 0.67 (10 µmol m^−2^ s^−1^ blue light, and 15 µmol m^−2^ s^−1^ green light) led to almost 70% reduction in stomatal opening [28]. Additionally, treatment with 1:1 blue and green photons (25 µmol m^−2^ s^−1^) has been shown to result in a 40% decrease in chloroplast avoidance movement (aggregation of chloroplasts along the anticlinal sides of the cell wall; 100% avoidance movement occurs under pure blue light), and treatment with a B:G ratio of 1.6 (25 µmol m^−2^ s^−1^ blue light, and 15 µmol m^−2^ s^−1^ green light) led to a ~ 70% reduction in avoidance movement in *Landoltia punctate* [26]. Taken together, only 5–10 µmol m^−2^ s^−1^ discrepancies between blue and green PPF are needed to alter physiological processes such as chloroplast positioning, stomatal opening, and ultimately rates of gas exchange. Therefore, ND filters may lead to over- or underestimation of whole-plant responses to foliar shade if used in simulations because they do not alter blue and green PPF as real world foliar shade does.

While the simulation of foliar shade by ND filters may not be completely realistic, having a degree of neutrality may be a good option for researchers who do not have a specific SPD simulation in mind and are more interested in reductions in the R:FR ratio, which ND filters can simulate well. Based on data we acquired under foliar shade, Rosco e-colour + ND filters would be best suited for future research, and only LEE Filters 0.90 ND would provide an adequate simulation of deep foliar shade spectral quality (Fig. 5; Table 3).

**Fig. 6:**
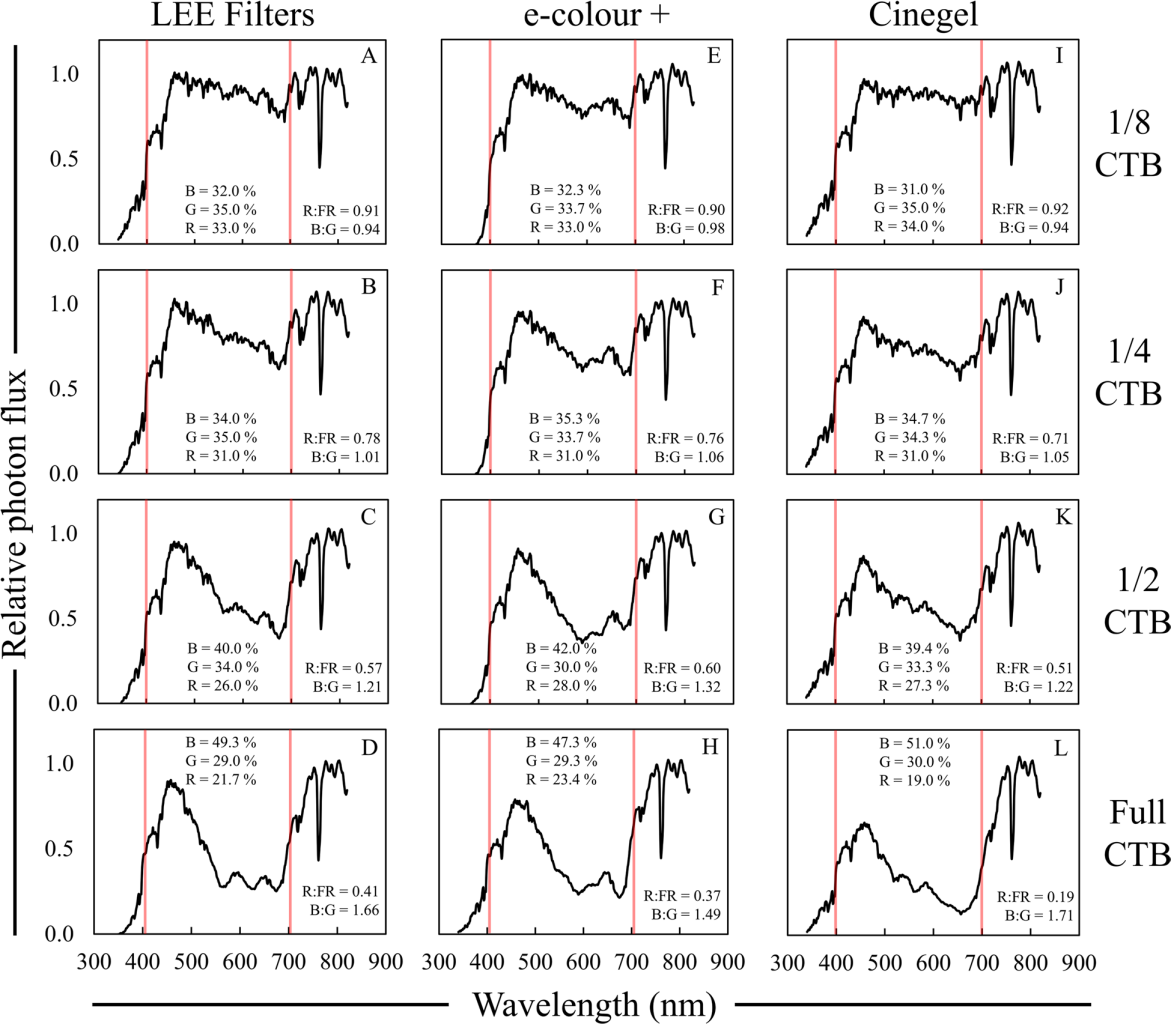
Relative spectral photon distributions (SPD) acquired under 1/8—full strength color temperature blue (CTB) filters from LEE Filters (A-D), Rosco e-colour + (**E**–**H**), and Rosco Cinegel (**I**–**L**). Data were normalized to the photon flux at 800 nm and are presented as the average of relative SPDs acquired on 27 May, 30 May, and 12 June 2020 on clear sky or mostly sunny days between 13:00–14:00 h. The average relative PPF of blue (B), green (G), and red (R) light are inset in each panel (see Additional file 2: Table S3 for sample statistics); the average R:FR (655–665 nm/725–735 nm) and B:G (420–490 nm/500–570 nm) ratios are inset in each panel. Data collected under full sun were on average; R:FR = 1.15, B:G = 0.87, % Blue = 29%, % Green = 35%, and % Red = 36%. Red bars indicate 400 and 700 nm, respectively, designating photosynthetically active radiation (PAR) between the red bars

Color temperature blue filters reduced the flux of photons with wavelengths between 550 and 700 nm to the greatest degree, with variation in the magnitude of the reduction being related to the strength of the filter (Fig. 6). Similar to ND filters, the strength of the CTB filter did not reflect the magnitude change in the R:FR ratio or the other ratios that we calculated (Table 4). Rosco Cinegel CTB filters reduced the R:FR ratio to a greater degree compared to LEE and Rosco e-colour + filters, which was also reflected in the PPE (Table 4). All CTB filters increased the B:G ratio and the percentage of blue light, similar to what was observed under moderate foliar shade (Fig. 4; Table 2), with varying effects depending on the strength of the filter (Table 4). However, only 1/4 CTB filters increased the B:G ratio to a range that was comparable to moderate foliar shade (Fig. 6A, E, I; Tables 2, 4).

**Table 4:** Average data collected under color temperature blue (CTB) photoselective filters

Filter	Brand	R:FR^a^	PPE^b^	B:G^c^	PPF reduction^d^	UV-A PF^e^
					%	µmol m^−2^ s^−1^
1/8 CTB	LEE	0.91 A^f^	0.69 A	0.94 I	20.7 G	58.5 A
	e-colour +	0.90 A	0.68 B	0.98 H	24.7 F	18.6 E
	Cinegel	0.92 A	0.70 A	0.94 I	24.3 F	63.2 A
1/4 CTB	LEE	0.78 B	0.67 C	1.01 G	28.3 E	48.4 BC
	e-colour +	0.76 BC	0.66 D	1.06 F	30.0 E	17.9 E
	Cinegel	0.71 C	0.67 C	1.05 F	35.0 D	59.7 A
1/2 CTB	LEE	0.57 D	0.63 E	1.21 E	44.0 C	38.2 D
	e-colour +	0.60 D	0.62 F	1.32 D	45.7 C	22.9 E
	Cinegel	0.51 E	0.63 E	1.22 E	45.7 C	54.9 AB
Full CTB	LEE	0.41 F	0.57 G	1.66 B	56.3 B	22.9 E
	e-colour +	0.37 F	0.56 H	1.49 C	57.7 B	42.3 CD
	Cinegel	0.19 G	0.51 I	1.71 A	67.7 A	43.0 CD

^a^R:FR = 655–665/725–735

^b^PPE = Phytochrome photoequilibria

^c^B:G = 420–490/500–570

^d^PPF reduction = Percent reduction in PPF relative to full sun

^e^UV-A PF = Photon flux between 340 and 399 nm

^f^Data are presented as averages acquired on three different clear sky or mostly sunny days between 13:00 and 14:00 h: 27 May, 30 May, and 12 June 2020. Means are only compared within column and were separated with Fisher’s LSD. Means followed by a common letter are not significantly different (*P* = 0.05). Data collected under full sun were on average; R:FR = 1.15, PPE = 0.72, B:G = 0.87, PPF = 1719 µmol m^−2^ s^−1^, UV-A PF = 86 µmol m^−2^ s^−1^

Rosco Cinegel CTB filters stood out specifically due to their ability to maintain the initial shape of the SPD with increasing filter strength, leading to a more “natural” change in spectral quality, primarily due to decreasing a broader range of wavelengths (Fig. 6). Color temperature blue filters exhibited significant differences in the quantity of UV-A light transmitted between the three filter brands, with Rosco e-colour + filters leading to the lowest UV-A transmission for 1/8–1/2 strength filters (Table 4). Overall, CTB filters transmitted a considerable amount of UV-A photons relative to data collected under foliar shade (Table 2), and in general UV-A photon flux decreased with increase in filter strength. However, Rosco e-colour + gels had the inverse effect on UV-A light, increasing the quantity of UV-A with increasing filter strength (Table 4). This may be due to the addition of different dyes or alterations in the ratios of the dyes in the filter as filter strength increases, rather than only an increase in dye concentration, leading to minor, yet unexpected changes in spectral quality.

While both LEE and Rosco e-colour + filters led to more synthetic changes in light quality as filter strength increased (Fig. 6). On their own, 1/4 CTB filters, and to some extent 1/2 CTB filters, provided a modest simulation of moderate foliar shade SPDs. The initial SPD shape produced by the CTB filter, especially 1/4 CTB filters, is well suited to simulate moderate foliar shade, but the R:FR ratio, the B:G ratios, and the UV-A photon flux were not in line with foliar shade data (Fig. 6B, F, J). The relatively high amount of UV-A photon flux observed under all CTB filters may inhibit shade avoidance responses brought on by the low R:FR ratios, and further reductions in UV-A photon flux with UV specific filters or plastics may further improve this system.

We are not aware of CTB filters being used in shade-based research, but other blue filters have been commonly used. Petrella and Watkins [40] and Studzinska et al. [44] used a blue polyethylene filter that only reduced the R:FR ratio to ~ 0.70 and did not provide an accurate simulation of moderate foliar shade due to a lack of a strong increase in the B:G ratio (Fig. 1; Table 1). Though not to specifically simulate foliar shade, Runkle and Heins [42] used a LEE Filters Peacock blue filter (Product No. 115) which reduced the R:FR in sunlight to 0.04, lower than any common type of foliar shade, and the overall SPD of the Peacock blue filter in sunlight does not accurately simulate any type of foliar shade (Fig. 1; Table 1). Hurdzan and Klein [38] used a Slate blue filter (Cinemoid 61) in combination with a Medium amber filter (Cinemoid 4) to simulate deciduous shade, resulting in a R:FR ratio of 0.75, with no mention of other ratios or the overall SPD. McVey and Mayer [62] were the first to report using blue-filtering materials to simulate foliar shade on an agricultural crop, Kentucky bluegrass (*Poa pratensis*), where they used blue acrylic plastic to alter spectral quality of sunlight in the field; no spectral ratios were provided, but SPD data that were provided showed that their treatment did not lead to an accurate simulation, and the strength of the alteration in light quality was unrealistic. Compared to this previous research, our data show that CTB filters provide much improved accuracy in regards to simulations of moderate foliar shade compared to blue filters used in previous research, but further reductions in UV light may be needed to prevent inhibition of shade avoidance responses as has been shown in previous research [29].

**Fig. 7:**
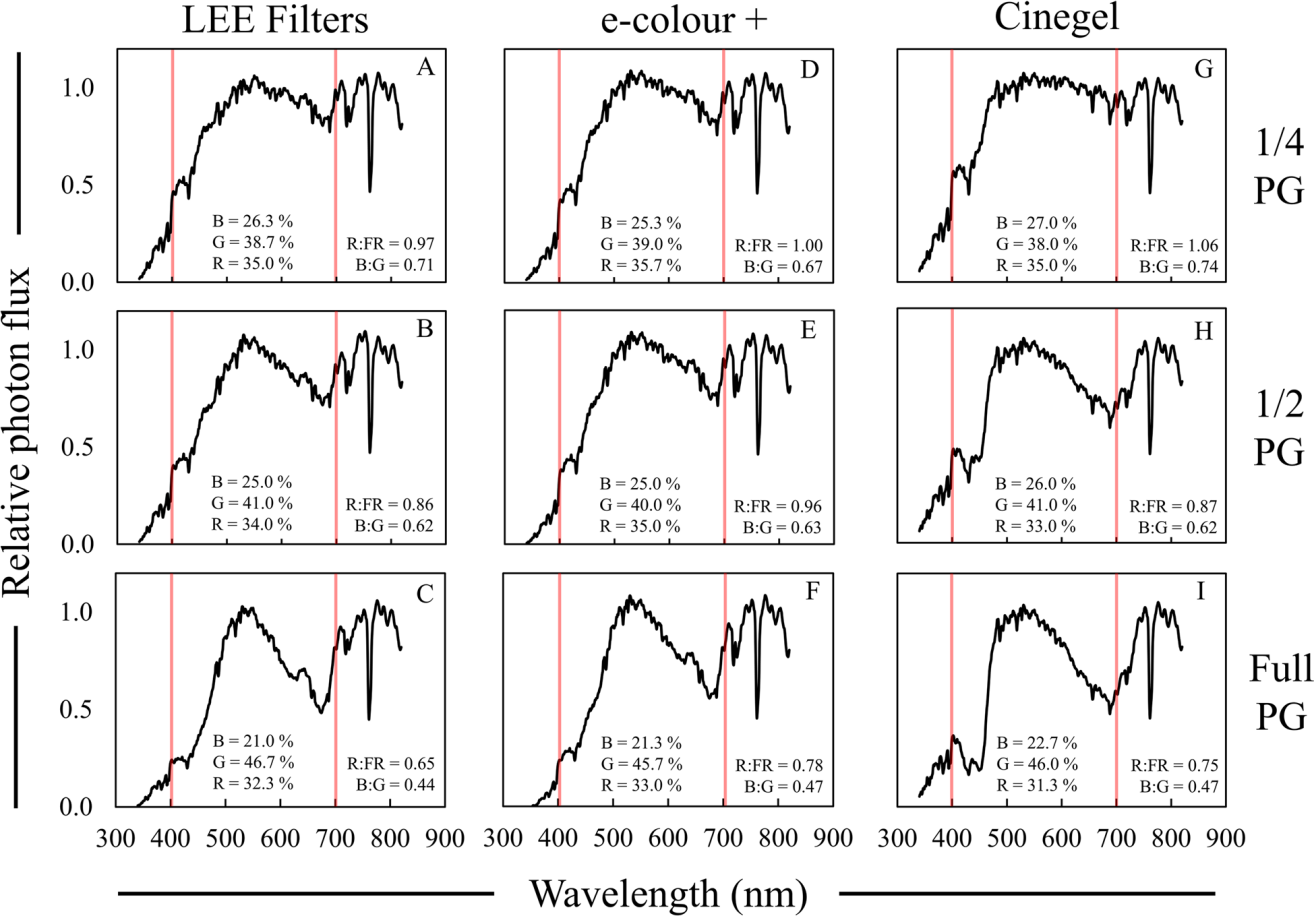
Relative spectral photon distributions (SPD) acquired under 1/4—full strength plus green (PG) filters from LEE Filters (**A**–**C**), Rosco e-colour + (**D**–**F**), and Rosco Cinegel (**G**–**I**). Data were normalized to the photon flux at 800 nm and are presented as the average of relative SPDs acquired on 27 May, 30 May, and 12 June 2020 on clear sky or mostly sunny days between 13:00–14:00 h. The average relative PPF of blue (B), green (G), and red (R) light are inset in each panel (see Additional file 2: Table S4 for sample statistics); the average R:FR (655–665 nm/725–735 nm) and B:G (420–490 nm/500–570 nm) ratios are inset in each panel. Data collected under full sun were on average; R:FR = 1.15, B:G = 0.87, % Blue = 29%, % Green = 35%, and % Red = 36%. Red bars indicate 400 and 700 nm respectively, designating photosynthetically active radiation (PAR) between the red bars

Plus green filters were evalated to determine how well they simulate deep foliar shade spectral motifs (Fig. 7). Both 1/4 and 1/2 PG filters led to B:G ratios that were comparable to what was observed under deep foliar shade (~ 0.53–0.75), and full PG filters resulted in to extreme of a reduction in the B:G ratio (Table 5). Other data, including the R:FR ratio the UV-A photon flux, were not comparable to what is observed under moderate or deep foliar shade.

**Table 5:** Average data collected under plus green (PG) photoselective filters

Filter	Brand	R:FR^a^	PPE^b^	B:G^c^	PPF reduction^d^	UV-A PF^e^
					%	µmol m^−2^ s^−1^
1/4 PG	LEE	0.97 AB^f^	0.70 C	0.71 AB	20.0 D	54.6 AB
	e-colour +	1.00 A	0.71 B	0.67 BC	19.0 D	39.1 CD
	Cinegel	1.06 A	0.71 A	0.74 A	17.3 D	67.9 A
1/2 PG	LEE	0.86 CD	0.69 D	0.62 C	25.7 C	47.8 BC
	e-colour +	0.96 ABC	0.70 BC	0.63 C	24.7 C	30.5 D
	Cinegel	0.87 BCD	0.70 C	0.62 C	27.3 C	66.9 A
Full PG	LEE	0.65 F	0.67 F	0.44 D	39.3 A	30.4 D
	e-colour +	0.78 DE	0.68 E	0.47 D	36.0 AB	13.4 E
	Cinegel	0.75 EF	0.69 D	0.47 D	34.7 B	58.1 AB

^a^R:FR = 655–665/725–735

^b^PPE = Phytochrome photoequilibria

^c^B:G = 420–490/500–570

^d^PPF reduction = Percent reduction in PPF relative to full sun

^e^UV-A PF = Photon flux between 340 and 399 nm

^f^Data are presented as averages acquired on three different clear sky or mostly sunny days between 13:00 and 14:00 h: 27 May, 30 May, and 12 June 2020. Means are only compared within column and were separated with Fisher’s LSD. Means followed by a common letter are not significantly different (*P* = 0.05). Data collected under full sun were on average; R:FR = 1.15, PPE = 0.72, B:G = 0.87, PPF = 1719 µmol m^−2^ s^−1^, UV-A PF = 86 µmol m^−2^ s^−1^

Green filters like PG have been used in previous research but do not provide accurate simulations of foliar shade on their own (Fig. 7). Hilton et al. [46] used green Cinemoid filters (Product No. 39) to examine the effects of reductions in the PPE on the germination of *Poa trivialis*, and while the authors stated that their filter treatment reduced the PPE to 0.03, no further spectral data were provided. Skálová and Krahulec [43] used a green filter that reduced the R:FR by only 31% compared to full sun, and the SPD of the green filter used did not provide an accurate simulation. Gautier et al. [35] used a LEE Filters Dark green filter (Product No. 124) that reduced the R:FR ratio to 0.04, and similar to the Peacock blue filter, the Dark green filter led to a strong alteration in light quality that was not very realistic (Fig. 1; Table 1). Pallas et al. [63] used green saran shade cloth of varying strength and showed that this material simulated foliar shade somewhat at lower strengths, but at higher strengths, led to mostly neutral PPF reductions. Overall, while PG filters provided more accurate simulations compared to other green filters previously used, green filters in general do not provide accurate simulations of deep foliar shade. These filters are less accurate than CTB filters overall, even if some spectral ratios match with data collected under foliage.

For ND, CTB, and PG filters there were differences in the overall SPD and specific spectral ratios between the brands we evaluated. Jackman [45] indicated that inconsistences can exist between brands even if filters, like ND, CTB, and PG, are considered industry standards, which could be seen when looking at the filters themselves (Fig. 2). The Rosco e-colour + line of filters is meant as an American version of LEE Filter’s European filters, and even with this, the two brands are different enough to lead to differences in important spectral ratios, especially with increased filter strength, without strong visual differences in the filters themselves (Fig. 2). Taken together, even if two filters look similar to the human eye, the way in which they alter light quality can be very different. If a researcher wanted to use, for example, a CTB filter, it would also be important to choose a specific brand rather than choosing a generic filter.

### Layering photoselective filters

Each filter had its own benefits and shortcomings when it came to more accurately simulating foliar shade; with that in mind, layering the filters to combine these benefits may further the ability to simulate foliar shade. Based on the results of the single filters, we moved forward with layering Rosco e-colour + ND, Rosco Cinegel CTB, and Rosco PG filters as well as LEE ND, CTB, and PG filters (LEE Filters data are presented in Additional file 1: Figs. S8–S11 and Additional file 2: Tables S8–S12).

**Fig. 8:**
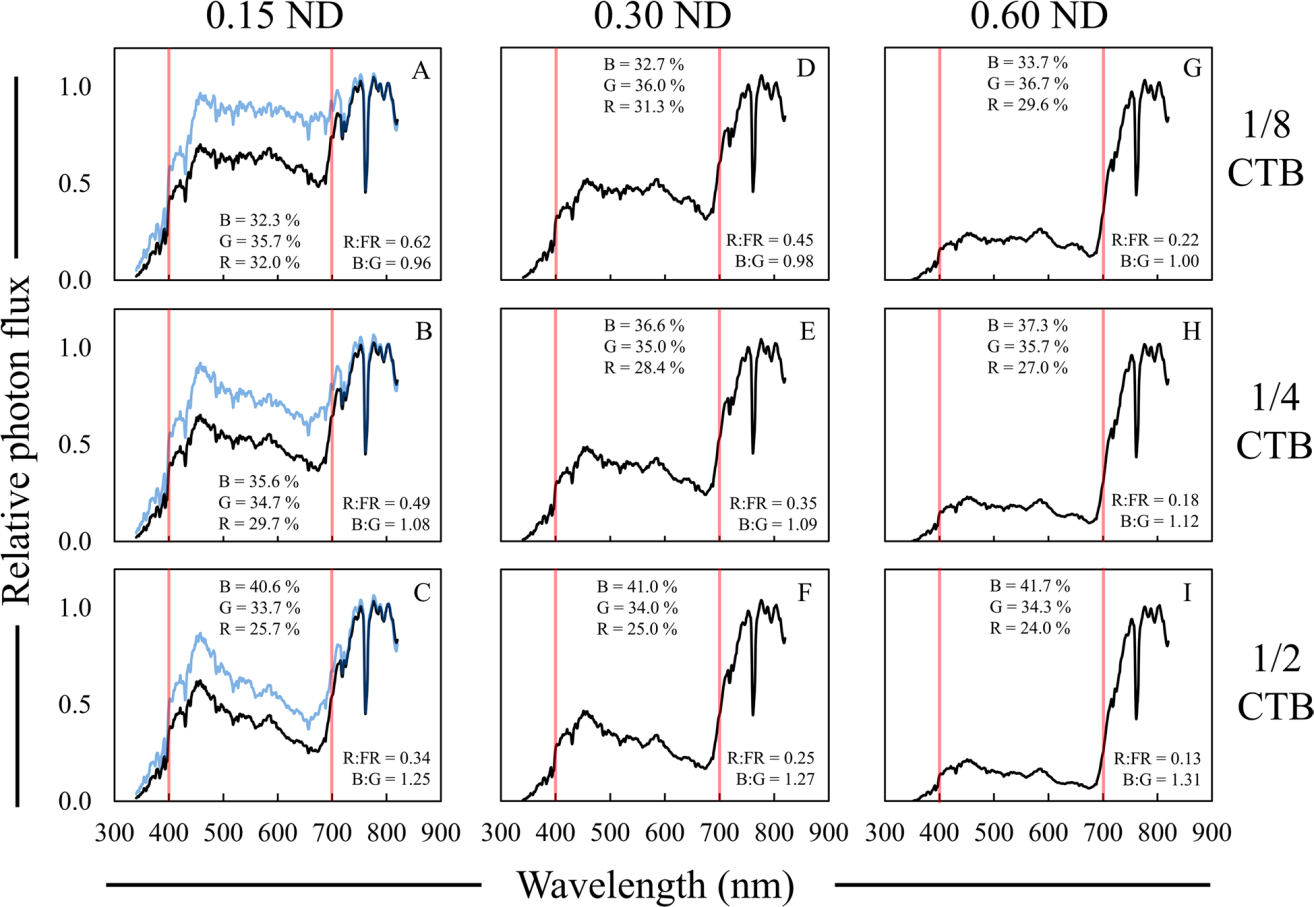
Relative spectral photon distributions (SPD) acquired under the combination of 1/s—1/2 Rosco Cinegel color temperature blue (CTB) and Rosco e-colour + 0.15–0.60 neutral density (ND) filters. **A**–**C** 0.15 ND + 1/8, 1/4, or 1/2 CTB. **D**–**F** 0.30 ND + 1/8, 1/4, or 1/2 CTB. **G**–**I** 0.60 ND + 1/8, 1/4, or 1/2 CTB. Data were normalized to the photon flux at 800 nm and are presented as the average of relative SPDs acquired on 27 May, 30 May, and 12 June 2020 on clear sky or mostly sunny days between 13:00 and 14:00 h. The average relative PPF of blue (B), green (G), and red (R) light are inset in each panel (see Additional file 2: Table S5 for sample statistics); the average R:FR (655–665 nm/725–735 nm) and B:G (420–490 nm/500–570 nm) ratios are inset in each panel. Data collected under full sun were on average; R:FR = 1.15, B:G = 0.87, % Blue = 29%, % Green = 35%, and % Red = 36%. Red bars indicate 400 and 700 nm, respectively, designating photosynthetically active radiation (PAR) between the red bars. Black lines represent the relative SPD of the layered filters, and blue lines represent the SPD of the CTB original filter

Layering CTB and ND filters improved upon the SPD and spectral ratios of the single filters (Fig. 8; Table 6). The addition of a ND filter on top of a CTB filter significantly reduced the R:FR ratio compared to either of these types of filters by themselves. Increasing strength of the ND filter also led to significant increases in the B:G ratio and the percent blue light for all CTB filters evaluated (Fig. 8; Table 6). The spectral shape and B:G ratio of the 1/4 CTB filter on its own simulated the SPD of moderate foliar shade well, but the R:FR was too high; with the addition of a ND filter, the R:FR ratio was reduced to a level that accurately simulated what we reported from the field (Tables 2, 6).

**Table 6:** Average data collected under layered color temperature blue (CTB) neutral density (ND) filters

Filter(s)	R:FR^a^	PPE^b^	B:G^c^	PPF reduction^d^	UV-A PF^e^
				%	µmol m^−2^ s^−1^
0.15 ND^f^	0.84 B^g^	0.68 B	0.86 K	36.0 K	45.5 A
0.30 ND	0.61 D	0.64 E	0.88 K	54.0 H	32.3 BCD
0.60 ND	0.30 H	0.53 J	0.91 J	79.7 C	12.5 HI
1/8 CTB	0.92 A	0.70 A	0.94 I	24.3 L	18.6 FGH
1/4 CTB	0.71 C	0.67 C	1.05 F	35.0 K	17.9 GH
1/2 CTB	0.51 E	0.63 F	1.22 C	45.7 J	22.9 EFG
**1/8 CTB**					
+ 0.15 ND	0.62 D	0.65 D	0.96 HI	48.7 I	37.3 AB
+ 0.30 ND	0.45 F	0.61 H	0.98 H	63.3 F	26.8 CDEF
+ 0.60 ND	0.22 I	0.49 K	1.00 G	83.7 B	9.9 HI
**1/4 CTB**					
+ 0.15 ND	0.49 E	0.62 G	1.08 E	57.7 G	34.6 BC
+ 0.30 ND	0.35 G	0.57 I	1.09 E	69.3 E	23.9 DEFG
+ 0.60 ND	0.18 J	0.45 L	1.12 D	86.7 A	9.0 I
**1/2 CTB**					
+ 0.15 ND	0.34 G	0.57 I	1.25 B	65.3 F	30.7 BCDE
+ 0.30 ND	0.25 I	0.52 J	1.27 B	74.7 D	23.6 DEFG
+ 0.60 ND	0.13 K	0.40 M	1.31 A	88.7 A	7.8 I

^a^R:FR = 655–665/725–735

^b^PPE = Phytochrome photoequilibria

^c^B:G = 420–490/500–570

^d^PPF reduction = Percent reduction in PPF relative to full sun

^e^UV-A PF = Photon flux between 340 and 399 nm

^f^Rosco Cinegel was used for CTB and Rosco e-colour + was used for ND filters

^g^Data are presented as averages acquired on three different clear sky or mostly sunny days between 13:00 and 14:00 h: 27 May, 30 May, and 12 June 2020. Means are only compared within column and were separated with Fisher’s LSD. Means followed by a common letter are not significantly different (*P* = 0.05). Data collected under full sun were on average; R:FR = 1.15, PPE = 0.72, B:G = 0.87, PPF = 1719 µmol m^−2^ s^−1^, UV-A PF = 86 µmol m^−2^ s^−1^

The combination of CTB and ND filters led to UV-A photon flux that was between the photon flux for either filter alone, except for when the strongest strength filters were combined (Table 6). This was not the case of layered CTB and ND LEE Filters in which case UV-A photon flux decreased more when the filters were layered then either filter alone (Additional file 2: Table S1). Jackman [45] previously stated that alterations to light quality under layered filters may not align with what is expected based on data from under single filters (i.e. two layers of 1/2 CTB) do not equal the changes that occur under full strength CTB). This may explain why UV-A photon flux under layered CTB and ND more closely resembles that of ND alone rather than exhibiting a reduction in magnitude that would align with effects of both single filters.

Spectral changes from both 1/4 and 1/2 CTB combined with 0.30 and 0.15 ND filters, respectively provided for the most accurate simulations of moderate foliar shade (Fig. 8C, E). While, the B:G ratio may be “too high” for 1/2 CTB + 0.15 ND, the percentages of blue and green light were quite similar to data collected under moderate foliar shade (~ 40 and 35%), but the UV-A photon flux was relatively high compared to moderate foliar shade (30.7 vs 12.74 µmol m^−2^ s^−1^). However, the LEE Filters 1/2 CTB combined with 0.15 or 0.30 ND led to alterations in spectral quality, including reduction in UV-A photon flux that was even more similar to foliar shade compared to Rosco filter combinations (Additional file 2: Table S1). The addition of a relative increase in blue light with this simulation may help better predict whole-plant responses to moderate foliar shade: a greater amount of blue light may result in greater stomatal opening and conductance and potentially higher rates of photosynthesis [59, 64].

**Fig. 9:**
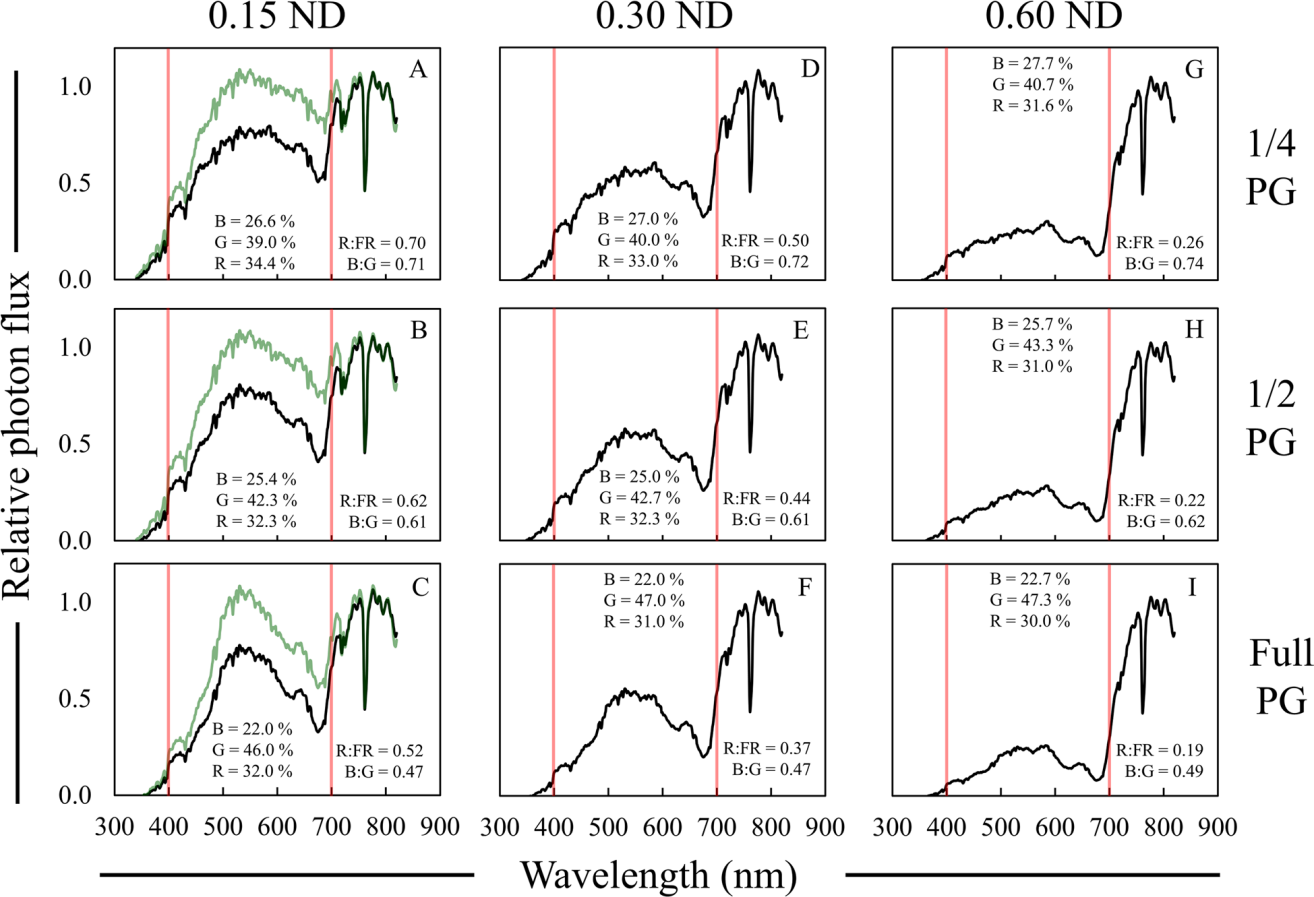
Relative spectral photon distributions (SPD) acquired under the combination of 1/4, 1/2, or full strength Rosco e-colour + plus green (PG) and Rosco e-colour + 0.15–0.60 neutral density (ND) filters. **A**–**C** 0.15 ND + 1/4, 1/2, or full strength PG. **D**–**F** 0.30 ND + 1/4, 1/2, or full strength PG. **G**–**I** 0.60 ND + 1/4, 1/2, or full strength PG. Data were normalized to the photon flux at 800 nm, and are presented as the average of relative SPDs acquired on 27 May, 30 May, and 12 June 2020 on clear sky or mostly sunny days between 13:00 and 14:00 h. The average relative PPF of blue (B), green (G), and red (R) light are inset in each panel (see Additional file 2: Table S6 for sample statistics); the average R:FR (655–665 nm/725–735 nm) and B:G (420–490 nm/500–570 nm) ratios are inset in each panel. Data collected under full sun were on average; R:FR = 1.15, B:G = 0.87, % Blue = 29%, % Green = 35%, and % Red = 36%. Red bars indicate 400 and 700 nm respectively, designating photosynthetically active radiation (PAR) between the red bars. Black lines represent the relative SPD of the layered filters, and green lines represent the SPD of the original PG filter

Layering ND filters on PG filters also improved upon the overall shade simulation of both filters alone (Fig. 9); however, in instances under foliar shade in the field where the B:G ratio is lower than that of full sun, the R:FR ratio was also generally low (Table 2). With that in mind, only 1/4 and 1/2 PG filters with 0.60 ND filters led to the more accurate simulations of deep foliar shade (Fig. 9; Table 7). Layered PG and ND filters also led to reductions in UV-A photon flux that were greater than either filter alone, but UV-A photon flux was only similar to data collected under foliar shade when a 0.60 ND filter was used. Overall, both 1/4 and 1/2 PG filters layered with 0.60 ND provided accurate simulations of deep foliar shade SPDs when the R:FR ratio looking to be simulated is > 0.20.

**Table 7:** Average data collected under layered plus green (PG) and neutral density (ND) filters

Filter(s)	R:FR^a^	PPE^b^	B:G^c^	PPF reduction^d^	UV-A PF^e^
				%	µmol m^−2^ s^−1^
0.15 ND^f^	0.84 B^g^	0.68 B	0.86 B	36.0 J	45.5 A
0.30 ND	0.61 D	0.64 E	0.88 AB	53.0 H	32.3 BC
0.60 ND	0.30 H	0.53 I	0.91 A	79.7 C	12.5 EF
1/4 PG	1.00 A	0.71 A	0.67 DE	19.0 L	39.1 AB
1/2 PG	0.96 A	0.70 A	0.63 EF	24.7 K	30.5 BC
Full PG	0.78 B	0.68 B	0.47 G	36.0 J	13.4 EF
**1/4 PG**					
+ 0.15 ND	0.70 C	0.67 C	0.71 CD	46.7 I	24.3 CD
+ 0.30 ND	0.50 E	0.62 F	0.72 C	61.7 F	17.4 DE
+ 0.60 ND	0.26 HI	0.50 J	0.74 C	83.0 B	7.1 FG
**1/2 PG**					
+ 0.15 ND	0.62 D	0.65 D	0.61 F	51.0 H	13.1 EF
+ 0.30 ND	0.44 F	0.60 G	0.61 F	66.7 E	10.6 EFG
+ 0.60 ND	0.22 IJ	0.48 K	0.62 F	84.3 AB	4.3 FG
**Full PG**					
+ 0.15 ND	0.52 E	0.63 E	0.47 G	56.7 G	8.8 EFG
+ 0.30 ND	0.37 G	0.58 H	0.47 G	69.7 D	6.4 FG
+ 0.60 ND	0.19 J	0.45 L	0.49 G	86.3 A	2.5 G

^a^R:FR = 655–665/725–735

^b^PPE = Phytochrome photoequilibria

^c^B:G = 420–490/500–570

^d^PPF reduction = Percent reduction in PPF relative to full sun

^e^UV-A PF = Photon flux between 340 and 399 nm

^f^Rosco e-colour + filters were used for both PG and ND filters

^g^Data are presented as averages acquired on three different clear sky or mostly sunny days between 13:00 and 14:00 h: 27 May, 30 May, and 12 June 2020. Means are only compared within column and were separated with Fisher’s LSD. Means followed by a common letter are not significantly different (*P* = 0.05). Data collected under full sun were on average; R:FR = 1.15, PPE = 0.72, B:G = 0.87, PPF = 1719 µmol m^−2^ s^−1^, UV-A PF = 86 µmol m^−2^ s^−1^

**Fig. 10:**
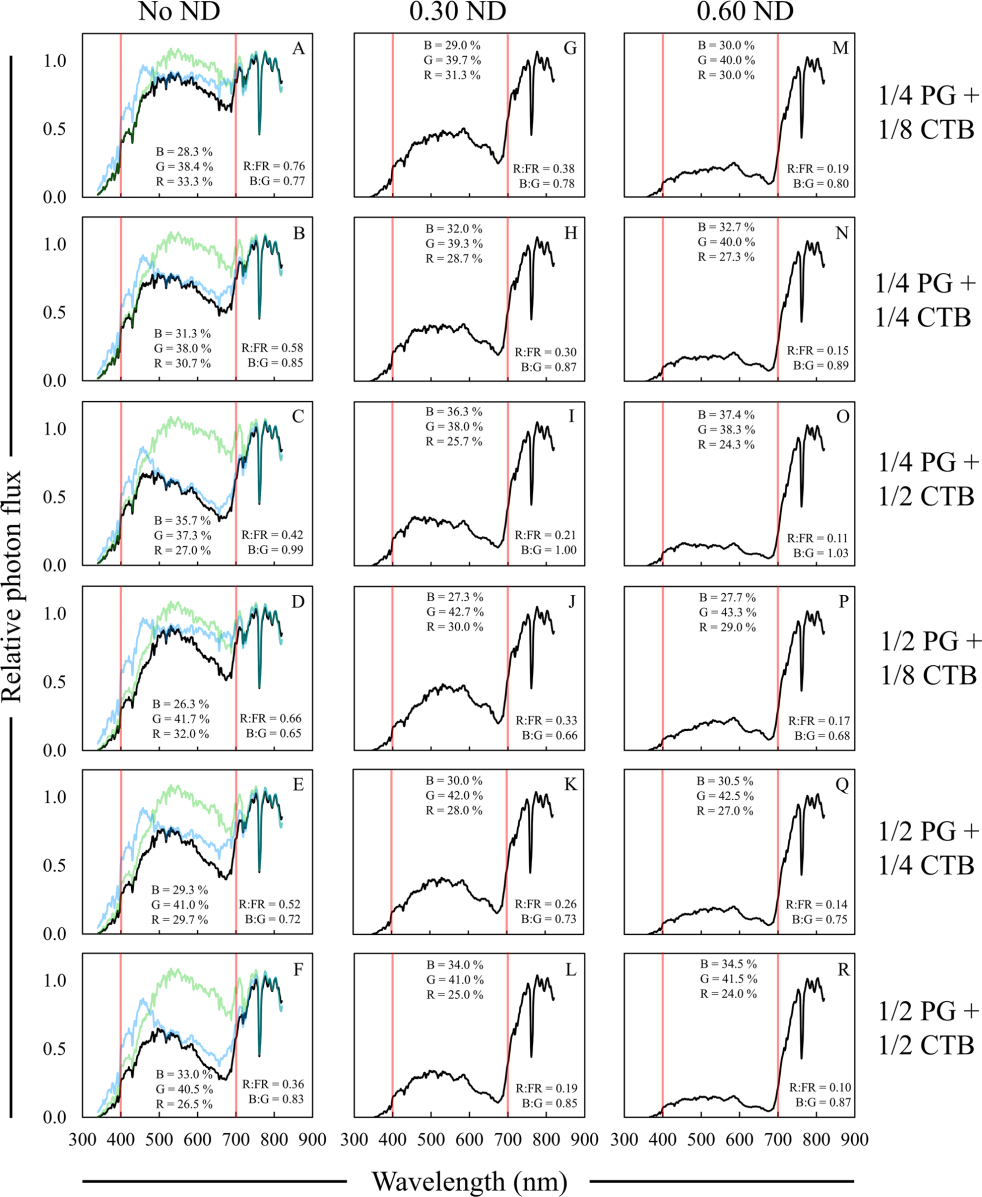
Relative spectral photon distributions (SPD) acquired under the combination of (1) 1/8, 1/4, or 1/2 Rosco Cinegel color temperature blue (CTB), (2) 1/4, 1/2, or full strength Rosco e-colour + plus green (PG), and (3) Rosco e-colour + 0.30 or 0.60 neutral density (ND) filters. **A**–**F** Combinations of CTB and PG filters only. **G**–**L** 0.30 ND filter layered on CTB + PG filters. **M**–**R** 0.60 ND filter layered on CTB + PG filters. Data were normalized to the photon flux at 800 nm and are presented as the average of relative SPDs acquired on 27 May, 30 May, and 12 June 2020 on clear sky or mostly sunny days between 13:00 and 14:00 h. The average relative PPF of blue (B), green (G), and red (R) light are inset in each panel (see Additional file 2: Table S7 for sample statistics); the average R:FR (655–665 nm/725–735 nm) and B:G (420–490 nm/500–570 nm) ratios are inset in each panel. Data collected under full sun were on average; R:FR = 1.15, B:G = 0.87, % Blue = 29%, % Green = 35%, and % Red = 36%. Red bars indicate 400 and 700 nm respectively, designating photosynthetically active radiation (PAR) between the red bars. Black lines represent the relative SPD of the layered filters, blue lines represent the SPD of the original CTB filter (**A**–**F**), green lines represent the SPD of the original PG filter (**A**–**F**)

Simultaneously layering ND, CTB, and PG filters resulted in the most accurate simulations of deep foliar shade SPDs (Fig. 10). Specifically, 1/s or 1/4 CTB filters combined with 1/2 PG and 0.60 ND filters led to the most accurate deep foliar shade simulation due to the overall SPD shape and the magnitude in the reduction of the R:FR ratio, B:G ratio, and the percentage of blue and green light in particular (Table 8, Fig. 10P, Q). Furthermore, UV-A photon flux was reduced to a quantity that was similar to data acquired under deep foliar shade sites in St. Paul MN when all three types of filters were layered.

**Table 8:** Average data collected under layered color temperature blue (CTB), plus green (PG), and neutral density (ND) filters

Filter(s)	R:FR^a^	PPE^b^	B:G^c^	PPF reduction^d^	UV-A PF^e^
				%	µmol m^−2^ s^−1^
**1/4 PG** **+** **1/8 CTB**^f^	0.76 A^g^	0.68 A	0.77 J	36.7 Q	36.4 A
+ 0.30 ND	0.38 F	0.58 F	0.78 I	69.7 K	15.1 D
+ 0.60 ND	0.19 JK	0.46 L	0.80 H	86.3 E	5.2 GH
**1/4 PG + 1/4 CTB**	0.58 C	0.65 C	0.85 F	46.3O	34.2 AB
+ 0.30 ND	0.30 H	0.55 H	0.87 E	74.0 I	14.1 D
+ 0.60 ND	0.15 LM	0.43 N	0.89 D	88.3 C	5.3 GH
**1/4 PG + 1/2 CTB**	0.42 E	0.61 E	0.99 C	55.7 M	31.6 B
+ 0.30 ND	0.21 J	0.50 J	1.00 B	79.0 G	12.4 DE
+ 0.60 ND	0.11 N	0.38 P	1.03 A	90.3 B	4.8 GH
**1/2 PG + 1/8 CTB**	0.66 B	0.67 B	0.65 P	43.1P	23.1 C
+ 0.30 ND	0.33 G	0.57 G	0.66 O	72.3 J	10.1 EF
+ 0.60 ND	0.17 KL	0.44 M	0.68 N	87.3 D	3.9 H
**1/2 PG + 1/4 CTB**	0.52 D	0.63 D	0.72 M	51.7 N	22.4 C
+ 0.30 ND	0.26 I	0.53 I	0.73 L	76.3 H	9.3 EF
+ 0.60 ND	0.14 M	0.41 O	0.75 K	89.0 C	3.8 H
**1/2 PG + 1/2 CTB**	0.36 F	0.59 F	0.83 G	61.1 L	21.1 C
+ 0.30 ND	0.19 K	0.48 K	0.85 F	81.3 F	8.3 FG
+ 0.60 ND	0.10 N	0.36 Q	0.87 E	91.3 A	2.9 H

^a^R:FR = 655–665/725–735

^b^PPE = Phytochrome photoequilibria

^c^B:G = 420–490/500–570

^d^PPF reduction = Percent reduction in PPF relative to full sun

^e^UV-A PF = Photon flux between 340 and 399 nm

^f^Rosco Cinegel was used for CTB and Rosco e-colour + was used for PG and ND filters

^g^Data are presented as averages acquired on three different clear sky or mostly sunny days between 13:00 and 14:00 h: 27 May, 30 May, and 12 June 2020. Means are only compared within column and were separated with Fisher’s LSD. Means followed by a common letter are not significantly different (*P* = 0.05). Data collected under full sun were on average; R:FR = 1.15, PPE = 0.72, B:G = 0.87, PPF = 1719 µmol m^−2^ s^−1^, UV-A PF = 86 µmol m^−2^ s^−1^

The R:FR ratio and percentages of blue and green light were in line with expectations for deep foliar shade for the combination of 1/2 CTB with 1/2 PG and either 0.30 or 0.60 ND filters, but this combination did not reduce the B:G ratio as low as what was observed under deep foliar shade sites in St. Paul, MN. Similarly, the use of 1/4 PG in the triple-layered combination did not lead to the more accurate simulated spectral reduction exhibited by the combination of 1/2 PG and 1/8 or 1/4 CTB filters (Table 8). Only the combination of 1/2 CTB and 1/4 PG with either 0.30 or 0.60 ND filters led to a more accurate simulation of moderate foliar shade; however, this did not improve simulation of moderate foliar shade compared to layering CTB and ND filters only.

Reducing the B:G ratio in combination with relatively large decrease in the R:FR ratio may exacerbate shade avoidance responses as well as lead to reductions in photosynthesis due to greater stomatal closure [28], especially if the difference between blue and green PPF ≥ 10 µmol m^−2^ s^−1^. However, researchers examining the effect of deep foliar shade may also be able to discover plants that utilize green wavelengths of light for photosynthesis more efficiently than other species at the whole-plant level when using layered filters rather than ND filters alone [23].

**Fig. 11:**
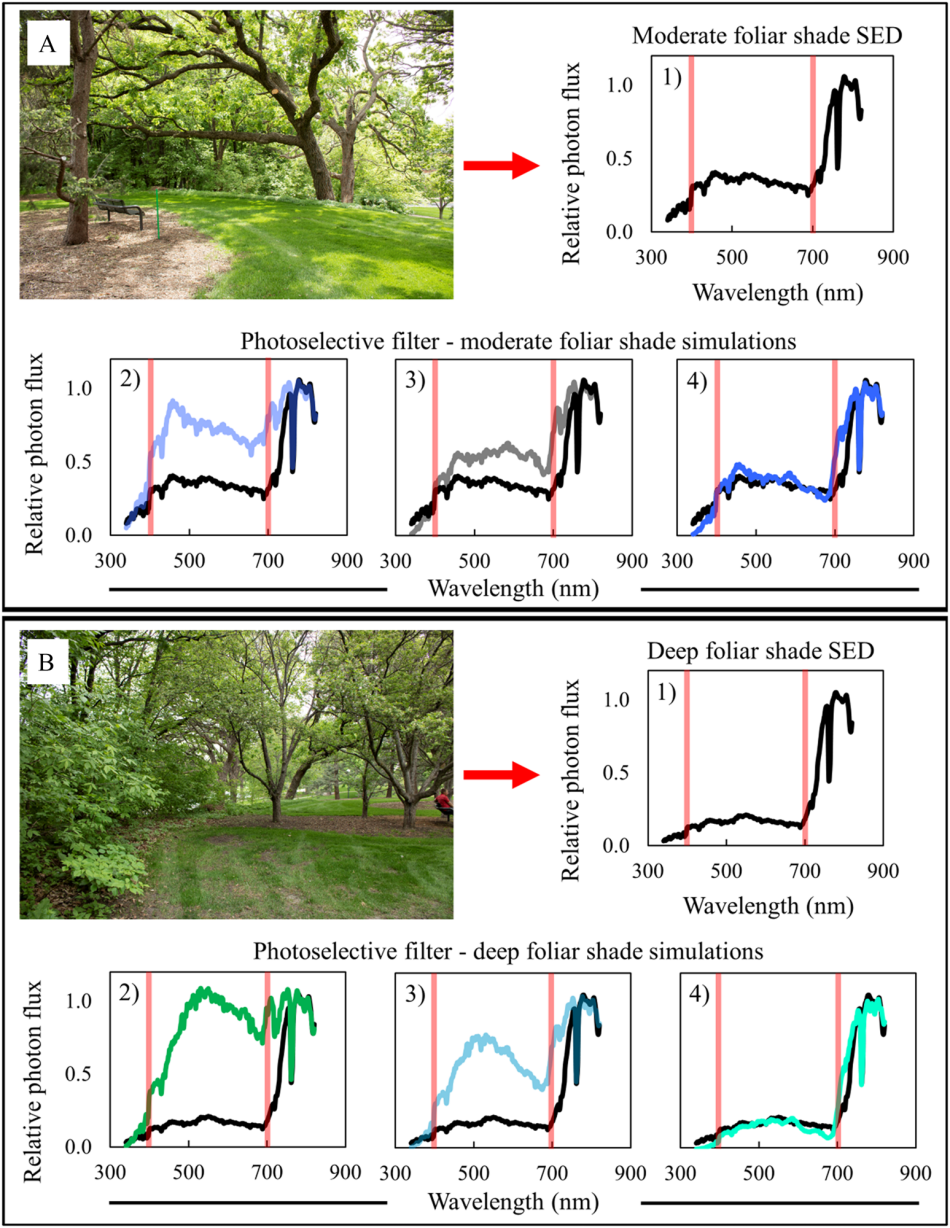
Two general models for simulating different types of foliar shade. In all panels, black lines in SPDs represent either moderate or deep foliar shade, and colored lines represent the SPD from the designated filter(s). Red bars indicate 400 and 700 nm, respectively, designating photosynthetically active radiation (PAR) between the red bars. **A** An area of moderate foliar shade with more diffuse light has an SPD with a (1) relatively high B:G ratio and a reduced R:FR ratio. A single Rosco Cinegel 1/4 CTB filter (2) simulates the B:G ratio of the SPD from the moderate foliar shade site, but does not simulate other parameters well. A single Rosco e-colour + 0.30 ND filter (3) similarly does not accurately simulate the moderate foliar shade SPD. The combination of the 1/4 CTB and the 0.30 ND filters (4) accurately simulates the entire moderate foliar shade SPD. **B** An area of deep foliar shade with a (1) relatively low B:G ratio and a much lower R:FR ratio. A single Rosco e-colour + 1/2 PG filter (2) does not simulate the deep shade foliar shade SPD. The combination of Rosco e-colour + 1/2 PG and a Rosco Cinegel 1/4 CTB filters (3) still does not provide an accurate deep foliar shade simulate. The combination of the PG, CTB, and a Rosco e-colour + 0.60 ND filter (4) accurately simulates the entire moderate deep shade SPD

The layering scheme that we have proposed could be tailored by researchers by mixing and matching different strengths of CTB, PG, and ND gels that best simulate selected spectra. Researchers can simulate a desired foliar shade SPD by first gathering spectral data in a target environment they hope to mimic, and then comparing the overall SPD shape and specific ratios like the R:FR and B:G ratios and percentages of specific wavelengths of PAR to those of single, double, and triple-layered filters to determine which is the most accurate (Fig. 11). With this method researchers will be able to generate accurate foliar shade simulations for both field and greenhouse experiments, eliminating the need for ND black shade cloth in foliar shade-related research.

### Photoselective filters and electric lighting

Because foliar shade simulations using filters would be advantageous in greenhouses, we next evaluated the effect of common greenhouse supplemental lighting on the spectral properties of the filters evaluated. To examine the maximum potential effect of supplemental lighting on spectral quality, we evaluated the SPD of HPS and quartz MH lamps under photoselective filters used in the triple-layered system at night when the lamps were the sole light source. UV-A photon flux was not quantified under sole-source supplemental lighting due to near zero UV-A light in the greenhouse at night.

Under HPS lamps, only 0.60 ND filters led to a reduction the R:FR ratio that was below what is normally observed under natural sunlight (~ 1.10–1.20), due to the synthetic spectra of the HPS lamp itself (Table 9). The combination of 0.60 ND and 1/2 CTB or 1/2 PG led to reductions in the R:FR ratio that were more in line with our foliar shade observations, but for all filters under HPS lamps, the PPE was at or near levels normally associated with full sun (Table 9). These differences in the R:FR ratio and the PPE may be due to the already altered spectral quality of the lamps and the small quantity of far-red light from the supplemental lamps in combination with the lower PPF output from the lamps [50]. Also, supplemental lighting has been previously indicated to have differential effects on the R:FR ratio and the PPE [65, 66]. The B:G ratios under all filters and all filter combinations also did not simulate foliar shade well under HPS lamps (Table 9).

**Table 9:** The effects of high-pressure sodium (HPS) lamps on filter spectral quality

Filter(s)	R:FR^a^	PPE^b^	B:G^c^	Blue^d^	Green^e^	Red^f^	PPF reduction^g^
				%			
Lamp only	3.45 A^i^	0.87 A	0.23 C	6.0 C	59.3 G	34.7 A	–
0.60 ND^h^	0.73 D	0.80 C	0.20 D	5.4 D	64.6 D	30.0 C	79.0 D
1/2 CTB	1.25 C	0.84 B	0.40 A	10.0 A	60.0 F	30.0 C	53.0 E
1/2 PG	2.47 B	0.87 A	0.14 E	5.1 D	62.6 E	32.3 B	22.3 F
0.60 ND + 1/2 CTB	0.28 F	0.73 E	0.29 B	7.4 B	66.6 C	26.0 E	90.3 B
0.60 ND + 1/2 PG	0.54 E	0.79 D	0.11 F	4.0 E	68.0 B	28.0 D	83.3 C
0.60 ND + 1/2 PG + 1/2 CTB	0.21 F	0.70 F	0.15 E	6.0 C	69.5 A	24.5 F	92.3 A

^a^R:FR = 655–665/725–735

^b^PPE = Phytochrome photoequilibria

^c^B:G = 420–490/500–570

^d^Blue = Percentage of PPF between 400 and 499 nm relative to total PPF

^e^Green = Percentage of PPF between 500 and 599 nm relative to total PPF

^f^Red = Percentage of PPF between 600 and 700 nm relative to total PPF

^g^PPF reduction = Percent reduction in PPF relative to full sun; PPF from HPS lamps only = 41 µmol m^−2^ s^−1^

^h^Rosco Cinegel was used for CTB and Rosco e-colour + was used for PG and ND filters

^i^Data are presented as averages acquired on three different days: 12 June, 13 June, and 14 June 2020 at 22:00 h in a greenhouse. Means are only compared within column and were separated with Fisher’s LSD. Means followed by a common letter are not significantly different (*P* = 0.05)

The high R:FR ratio produced by HPS lamps could by itself influence plant growth and development. The R:FR ratio is naturally reduced below 1.0 at both the end of the day and in the early morning (~ 0.60–0.80, depending on latitude) under full sun conditions [67]. Reductions in the R:FR ratio during the end of the day alter gene expression and lead to changes in shade avoidance like responses, regardless of daytime reductions in the R:FR ratio [68, 69]. Along the same lines, alterations in the R:FR ratio in the morning or the end of the day could interfere with expression of circadian regulated genes [70]. Therefore, the lack of a normal reduction in the R:FR ratio at the end of the day or early in the morning could influence research materials that receive supplemental HPS lighting.

Under MH lamps, spectral ratios were more similar to data we acquired under the filters in natural sunlight (Table 10). The reductions in R:FR ratio data were more extreme for the layered filters, but overall, the R:FR ratios of 0.60 ND and CTB filters provided more accurate simulations of foliar shade. The PPE under MH lamps was higher than what was observed from the field, but under the layered filters, the PPE was lower than ambient sunlight (~ 0.72). The B:G ratios of the filters under MH lamps were also improved. The CTB filter alone or in combination with ND filters did not increase the B:G ratio above 1.0 like it did under sunlight, but it was more elevated compared to HPS lamps. Similarly, the B:G ratios of the triple-layered filters were more reduced and in line with expectations with the B:G ratio under deep foliar shade (Table 9).

**Table 10:** The effects of metal halide (MH) lamps on filter spectral quality

Filter(s)	R:FR	PPE	B:G	Blue^d^	Green^e^	Red^f^	PPF reduction^g^
				%			
Lamp only	1.44 A^i^	0.80 B	0.55 E	24.0 D	63.0 C	13.0 A	–
0.60 ND^h^	0.40 D	0.72 D	0.63 C	26.0 C	63.0 C	11.0 C	77.0 D
1/2 CTB	0.57 C	0.74 C	0.82 B	33.5 B	57.0 D	9.5 E	44.0 E
1/2 PG	1.24 B	0.81 A	0.35 G	17.7 F	70.3 A	12.0 B	23.7 F
0.60 ND + 1/2 CTB	0.18 F	0.64 E	0.93 A	35.3 A	56.7 D	8.0 F	87.0 B
0.60 ND + 1/2 PG	0.32 E	0.71 D	0.40 F	19.0 E	70.7 A	10.3 D	82.3 C
0.60 ND + 1/2 PG + 1/2 CTB	0.15 F	0.63 F	0.58 D	26.0 C	66.0 B	8.0 A	90.0 A

^a^R:FR = 655–665/725–735

^b^PPE = Phytochrome photoequilibria

^c^B:G = 420–490/500–570

^d^Blue = Percentage of PPF between 400 and 499 nm relative to total PPF

^e^Green = Percentage of PPF between 500 and 599 nm relative to total PPF

^f^Red = Percentage of PPF between 600 and 700 nm relative to total PPF

^g^PPF reduction = Percent reduction in PPF relative to full sun; PPF from MH lamps only = 30 µmol m^−2^ s^−1^

^h^Rosco Cinegel was used for CTB and Rosco e-colour + was used for PG and ND filters

^i^Data are presented as averages acquired on three different days: 12 June, 13 June, and 14 June 2020 at 22:00 h in a greenhouse. Means are only compared within column and were separated with Fisher’s LSD. Means followed by a common letter are not significantly different (*P* = 0.05)

Overall, MH lamps maintained desired levels of specific ratios like the R:FR and B:G ratios relative to data from the field, and the ratios simulated foliar shade better than HPS lamps. These results represent the maximum potential change in spectral quality due to supplemental lighting, and minor modifications could be expected if the lighting is on during daytime hours. The PPE was higher under the filters, even with reduced R:FR ratios, and because of this, the altered spectral quality of the filter may have less of an effect on plant growth and development, as the PPE and the relative amount of far-red absorbing phytochrome (P_fr_) are better correlated to plant responses compared to the R:FR ratio [65]. This effect was lessened with MH lamps, but the relatively higher PPE may lead to less dramatic shade avoidance symptoms on the plants being tested.

## Conclusions

Our results showed that not all filters lead to accurate simulations of foliar shade, and that layering combinations of filters with contrasting qualities can produce an accurate simulation of different types of foliar shade, including spectral motifs of moderate and deep foliar shade such as reductions in the R:FR ratio, alterations in the B:G ratio, and changes in the proportion of blue and green PPF relative to the total PPF (Table 11). However, many filters or filter combinations did not reduce UV-A photon flux as much as what was observed under foliar shade sites. We hypothesize that the same would be observed with UV-B light, which has been shown to specially inhibit shade avoidance responses. While ND filters simulate reductions in the R:FR well, these filters do not alter blue and green light to the same degree in which foliar shade does. Small changes in blue and green PPF have been shown to alter plant physiology, stomatal biology in particular, to warrant the need to simulate these changes in whole-plant experiments. Therefore, combinations of CTB + ND, PG + ND, or CTB + PG + ND photoselective filters offer the best solution to examine holistic responses to foliar shade that take into account the effects from multiple wavelengths of light under one treatment.

**Table 11:** Combinations of color temperature blue (CTB), plus green (PG), and neutral density (ND) photoselective filters that best reflect moderate and foliar shade spectral motifs

Foliar shade simulation	Filter combination	Manufacturer (Model#)	R:FR^a^	B:G^b^	Blue^c^	Green^d^	Red^e^	PPF reduction^f^	UV-A PF^g^
					%				µmol m^−2^ s^−1^
**Moderate**	1/4 CTB +	Rosco Cinegel (#203) (LEE Filters #203)							
	0.30 ND	Rosco e-colour + (#3402) (LEE Filters #209)	0.35 (0.42)^h^	1.09 (0.97)	36.6 (33.0)	35 (36.0)	28.4 (31.0)	69.3 (66.0)	23.9 (16.3)
	1/2 CTB +	Rosco Cinegel (#202) (LEE Filters #202)							
	0.15 ND	Rosco Cinegel (#3415) (LEE Filters #298)	0.34 (0.42)	1.25 (1.18)	40.6 (39.7)	33.7 (34.0)	25.7 (26.3)	65.3 (61.3)	30.7 (21.9)
**Deep**	1/4 PG +	Rosco e-colour + (#3316) (LEE Filters #246)							
	0.60 ND	Rosco e-colour + (#3403) (LEE Filters #210)	0.26 (0.29)	0.74 (0.64)	27.7 (23.6)	40.7 (39.7)	31.6 (36.7)	83.0 (81.7)	7.1 (5.6)
	1/2 PG +	Rosco e-colour + (#3315) (LEE Filters #245)							
	0.60 ND	Rosco e-colour + (#3403) (LEE Filters #210)	0.22 (0.26)	0.62 (0.56)	25.7 (22.7)	43.3 (42.0)	31.0 (35.3)	84.3 (83.7)	4.3 (4.7)
	1/8 CTB +	Rosco Cinegel (#218) (LEE Filters #218)							
	1/2 PG +	Rosco e-colour + (#3315) (LEE Filters #245)							
	0.60 ND	Rosco e-colour + (#3403) (LEE Filters #210)	0.17 (0.20)	0.68 (0.60)	27.7 (24.4)	38.3 (42.3)	24.3 (33.3)	87.3 (85.3)	3.9 (4.7)
	1/4 CTB +	Rosco Cinegel (#203) (LEE Filters #203)							
	1/2 PG +	Rosco e-colour + (#3315) (LEE Filters #245)							
	0.60 ND	Rosco e-colour + (#3403) (LEE Filters #210)	0.14 (0.18)	0.75 (0.65)	30.5 (26.7)	42.5 (42.0)	27 (31.3)	89 (86.3)	3.8 (4.0)
	1/2 CTB +	LEE Filters (#202)							
	1/2 PG +	LEE Filters (#245)							
	0.60 ND	LEE Filters (#210)	0.13	0.78	31.6	40.7	27.7	89.30	3.30

Moderate foliar shade can be described as shade that exhibits a B:G ratio above 1.0 (36–40% blue PPF and 35% green PPF), a R:FR ratio that is typically ≥ 0.30, and reductions in PPF between 70–90%. Deep foliar shade can be described as shade that exhibits a B:G ratio below 0.80 (20–30% blue PPF and ≥ 40% green PPF) a R:FR ratio that is typically ≤ 0.20, and reductions in PPF ≥ 95%

^a^R:FR = 655–665/725–735

^b^B:G = 420–490/500–570

^c^Blue = Percentage of PPF between 400 and 499 nm relative to total PPF

^d^Green = Percentage of PPF between 500 and 599 nm relative to total PPF

^e^Red = Percentage of PPF between 600 and 700 nm relative to total PPF

^f^PPF reduction = Percent reduction in PPF relative to full sun

^g^UV-A PF = Photon flux between 340 and 399 nm

^h^Data for LEE Filters combinations are in parentheses

Interestingly, increasing strength of all filter types did not result in linear changes in spectral quality, and the same model filters from different brands did not lead to the same exact changes in spectral quality. In our study, Rosco filters, e-colour + and Cinegel, provided a more accurate simulation of moderate and deep foliar shade spectra collected in St. Paul MN compared to LEE Filters, but LEE Filters may similarly be more accurate for others’ collected spectra. Photoselective filters can be used in the field or in greenhouses to provide foliar shade simulations, but in greenhouses, supplemental lighting will further alter spectral quality. However, the use of MH supplemental lighting can help to limit these effects compared to HPS lamps. Simulating spectral ratios and SPDs of foliar shade, such as deep foliar shade, are especially important for advancing agroforestry and intercropping research, in which researchers are currently aiming to sustainably maximize yield in highly shaded environments [42, 43]. This is equally as important to help researchers simulate light found within forests to further understanding of forest ecology. Additionally, simulating deep and moderate foliar shade using the layered photoselective we have described can help plant breeders improve the selection of shade adapted plants, such as turfgrasses that are more fit for foliar shade. We have shown the ability to re-create foliar shade spectral quality using layered photoselective filters, something that cannot be done using neutral density shade cloth. This approach can be used to further our understanding of plant responses to foliar shade as well as improve the breeding of plants for shaded environment.

## Supplementary Information

**Additional file 1**: **Figure S1.** Spectroradiometer setup to collect spectral data under photoselective filters. A box with a ~ 3.0 x 3.0 cm hole was placed over the spectroradiometer in order to only expose the sensor to sunlight passed through a given photoselective filter. **Figure S2.** Foliar shade sites at the University of Minnesota St. Paul campus where SPD data were collected in 2018 (1-4, and 6) and in 2020 (5). 1) Maple grove-southern row, 2) Oak grove, 3) Northern forest edge, 4) Southern forest edge, 5) Maple grove-northern row, and 6) Within a forest. Satellite images were acquired through Google Earth at 1250 ft. Pictures of shade sites were adjusted for brightness and contrast to make foliage more visible. Stars indicate the approximate position data were acquired. **Figure S3.** Crop stands in which spectral data collected at the soil surface. A) Wheat field on 30 June 2018 (data were collected on 2 and 6 July 2018). B) Canola on 23 April 2018. C) Barley on 23 April 2018. **Figure S4.** Results of a linear regression between the narrow- and broadband R:FR ratio for data collected under full sun and under foliar shade in the field. Data within the light red shading fall within a 95% confidence interval for value prediction. **Figure S5.** Results of a linear regression between the narrow- and broadband B:G ratios for A) data collected under full sun and under foliar shade in the field, and B) data collected under foliar shade sites only. The red arrow indicates data collected under full sun. Data within the light blue shading fall within a 95% confidence interval for value prediction. **Figure S6.** Results of a linear regression between the narrow- and broadband A) R:FR ratios and B) B:G ratios for all data collectced under single and layered photoselective filters under natural light. The red arrows indicate data collected under Rosco e-colour+ 1/2 and Full CTB filters and LEE Filters full CTB filters. Data within the light red or blue shading fall within a 95% confidence interval for value prediction. **Figure S7.** Results of a linear regression between the narrow- and broadband A) R:FR ratios and B) B:G ratios for all data collectced under single and layered photoselective filters in the greenhouse under supplemental lighting. The red arrows indicates data collected under 1/2 and LEE Filters and Rosco e-colour+ Full CTB filters under HPS lamps Data within the light red or blue shading fall within a 95% confidence interval for value prediction. **Figure S8.** Relative spectral photon distributions (SPD) acquired under the combination of 1/8 – 1/2 LEE Filters color temperature blue (CTB) and LEE Filters 0.15 – 0.60 neutral density (ND) filters. A-C) 0.15 ND + 1/8, 1/4, or 1/2 CTB. D-F) 0.30 ND + 1/8, 1/4, or 1/2 CTB. G-I) 0.60 ND + 1/8, 1/4, or 1/2 CTB. Data were normalized to the photon flux at 800 nm and are presented as the average of relative SPDs acquired on 27 May, 30 May, and 12 June 2020 on clear sky or mostly sunny days between 13:00-14:00 h. Data collected under full sun were on average; R:FR = 1.15, B:G = 0.87, % Blue = 29%, % Green = 35%, and % Red = 36%. Red bars indicate 400 and 700 nm respectively, designating photosynthetically active radiation (PAR) between the red bars. Black lines represent the relative SPD of the layered filters, and blue lines represent the SPD of the original CTB filter. **Figure S9.** Relative spectral photon distributions (SPD) acquired under the combination of 1/4, 1/2, or full strength LEE Filters plus green (PG) and LEE Filters 0.15 – 0.60 neutral density (ND) filters. A-C) 0.15 ND + 1/4, 1/2, or full strength PG. D-F) 0.30 ND + 1/4, 1/2, or full strength PG. G-I) 0.60 ND + 1/4, 1/2, or full strength PG. Data were normalized to the photon flux at 800 nm, and are presented as the average of relative SPDs acquired on 27 May, 30 May, and 12 June 2020 on clear sky or mostly sunny days between 13:00-14:00 h. Data collected under full sun were on average; R:FR = 1.15, B:G = 0.87, % Blue = 29%, % Green = 35%, and % Red = 36%. Red bars indicate 400 and 700 nm respectively, designating photosynthetically active radiation (PAR) between the red bars. Black lines represent the relative SPD of the layered filters, and green lines represent the SPD of the original PG filter. **Figure S10.** Relative spectral photon distributions (SPD) acquired under the combination of 1) 1/8, 1/4, or 1/2 LEE Filters color temperature blue (CTB), 2) 1/4, 1/2 , or full strength LEE Filters plus green (PG), and 3) LEE Filters 0.30 or 0.60 neutral density (ND) filters. A-F) Combinations of CTB filters only. G-L) 0.30 ND filter layered on CTB + PG filters. M-R) 0.60 ND filter layered on CTB + PG filters. Data were normalized to the photon flux at 800 nm and are presented as the average of relative SPDs acquired on 27 May, 30 May, and 12 June 2020 on clear sky or mostly sunny days between 13:00-14:00 h. Data collected under full sun were on average; R:FR = 1.15, B:G = 0.87, % Blue = 29%, % Green = 35%, and % Red = 36%. Red bars indicate 400 and 700 nm respectively, designating photosynthetically active radiation (PAR) between the red bars. Black lines represent the relative SPD of the layered filters, blue lines represent the SPD of the original CTB filer (A-F), green lines represent the SPD of the original PG filter (A-F), and teal lines represent the SPD of layered CTB and PG filters (G-R). **Figure S11.** Two general models for simulating different types of foliar shade. In all panels, black lines in SPDs represent either moderate or deep foliar shade, and colored lines represent the SPD from the designated filter(s). Red bars indicate 400 and 700 nm respectively, designating photosynthetically active radiation (PAR) between the red bars. A) An area of moderate foliar shade with more diffuse light has an SPD with a (1) relatively high B:G ratio and a reduced R:FR ratio. A LEE Filters 1/4 CTB filter (2) simulates the B:G ratio of the SPD from the moderate foliar shade site, no other parameters are simulated well. A single LEE Filters 0.30 ND filter (3) similarly does not accurately simulate the moderate foliar shade SPD. The combination of the 1/4 CTB and the 0.30 ND filters (4) more accurately simulates the entire moderate foliar shade SPD. B) An area of deep foliar shade with a (1) relatively low B:G ratio and a much lower R:FR ratio. A single LEE Filters 1/2 PG filter (2) does not simulate the deep shade foliar shade SPD. The combination of a LEE Filters 1/2 PG and a LEE Filters 1/4 CTB filters (3) still does not provide an accurate deep foliar shade simulate. The combination of the PG, CTB, and a LEE Filters 0.60 ND filter (4) more accurately simulates the entire moderate deep shade SPD.

**Additional file 2**: **Table S1**: Average relative blue, green and red PPF data collected under either full sun or foliar shade. **Table S2**: Average relative blue, green and red PPF data collected under neutral density (ND) photoselective filters. **Table S3**: Average relative blue, green and red PPF data collected under color temperature blue (CTB) photoselective filters. **Table S4**: Average relative blue, green and red PPF data collected under plus green (PG) photoselective filters. **Table S5**: Average relative blue, green and red PPF data collected under layered Rosco color temperature blue (CTB) neutral density (ND) filters. **Table S6**: Average relative blue, green and red PPF data collected under layered Rosco plus green (PG) and neutral density (ND) filters. **Table S7**: Average relative blue, green and red PPF data collected under layered Rosco color temperature blue (CTB), plus green (PG), and neutral density (ND) filters. **Table S8**: Average data collected under layered LEE Filters color temperature blue (CTB) and neutral density (ND) filters. **Table S9**: Average data collected under layered LEE Filters plus green (PG) and neutral density (ND) filters. **Table S10**: Average data collected under layered LEE Filters color temperature blue (CTB), plus green (PG), and neutral density (ND) filters. **Table S11**: The effects of high-pressure sodium (HPS) lamps on LEE Filters spectral quality. **Table S12**: The effects of metal halide (MH) lamps on LEE Filters spectral quality.

## Data Availability

Data will be made available on acceptance of this manuscript at the University of Minnesota digital conservancy Data Repository of U of M (DRUM).

## Abbreviations

B: Blue
B:G: Blue to green
CTB: Color temperature blue
FAD: Flavin adenine dinucleotide
FMN: Flavin mononucleotide
G: Green
HPS: High-pressure sodium
LED: Light emitting diode
MH: Metal halide
ND: Neutral density
Pfr: Far-red absorbing phytochrome
Pr: Red absorbing phytochrome
PF: Photon flux
PPF: Photosynthetic photon flux
PAR: Photosynthetically active radiation
PPE: Phytochrome photoequilibria
PG: Plus green
R: Red
R:FR: Red to far-red
UV: Ultraviolet
UVR8: UV resistance locus 8
