# Supplementary material for: Layering contrasting photoselective filters improves the simulation of foliar shade
Source: Plant Methods. 2022 Feb 8;18:16. doi: 10.1186/s13007-022-00844-8 (PMC8822638; doi:10.1186/s13007-022-00844-8)

**Supplemental Figure S1:** Spectroradiometer setup to collect spectral data under photoselective filters. A box with a ~ 3.0 x 3.0 cm hole was placed over the spectroradiometer in order to only expose the sensor to sunlight passed through a given photoselective filter.


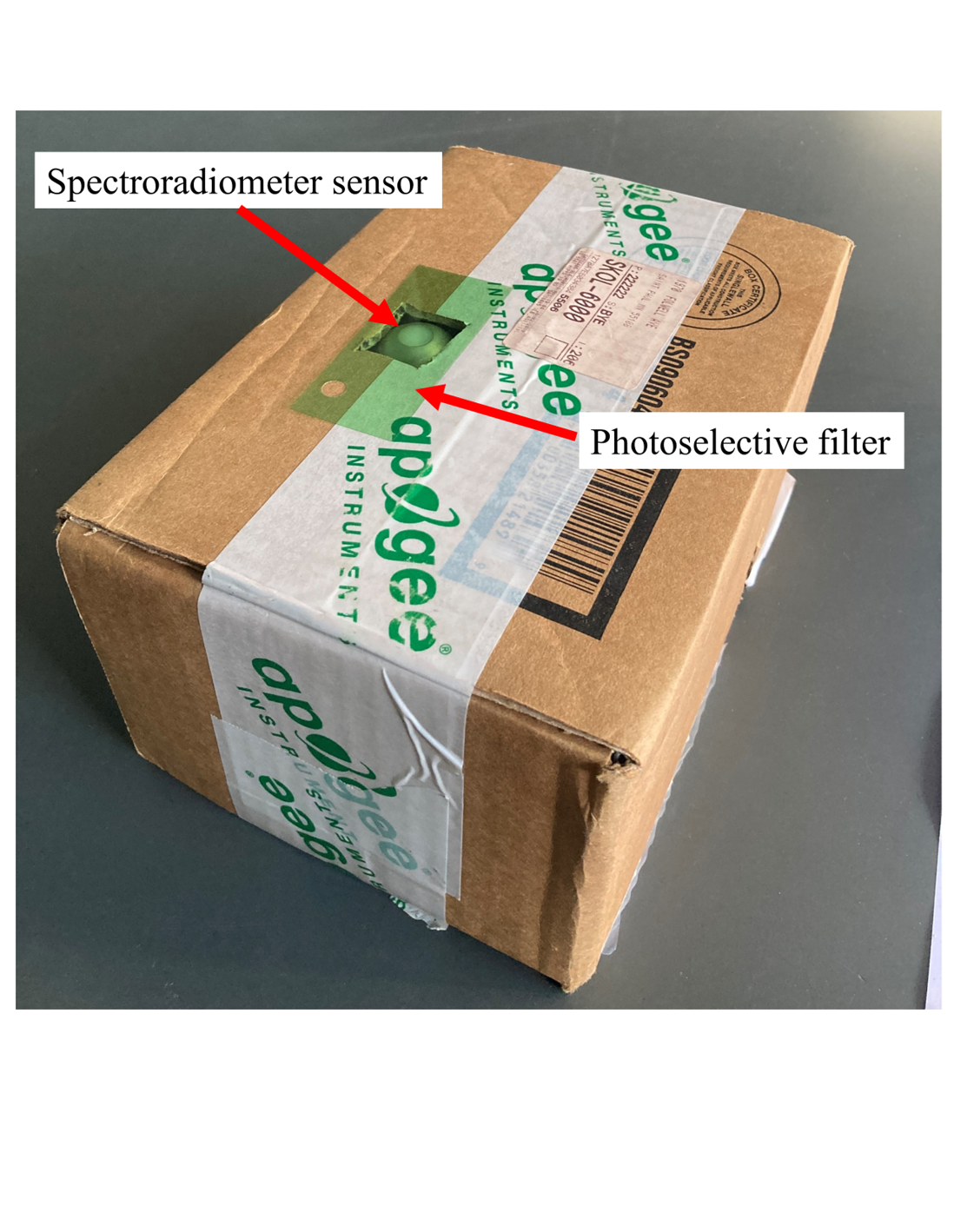


**Supplemental Figure S2:** Foliar shade sites at the University of Minnesota St. Paul campus where SPD data were collected in 2018 (1-4, and 6) and in 2020 (5). 1) Maple grove-southern row, 2) Oak grove, 3) Northern forest edge, 4) Southern forest edge, 5) Maple grove-northern row, and 6) Within a forest. Satellite images were acquired through Google Earth at 1250 ft. Pictures of shade sites were adjusted for brightness and contrast to make foliage more visible. Stars indicate the approximate position data were acquired.

**
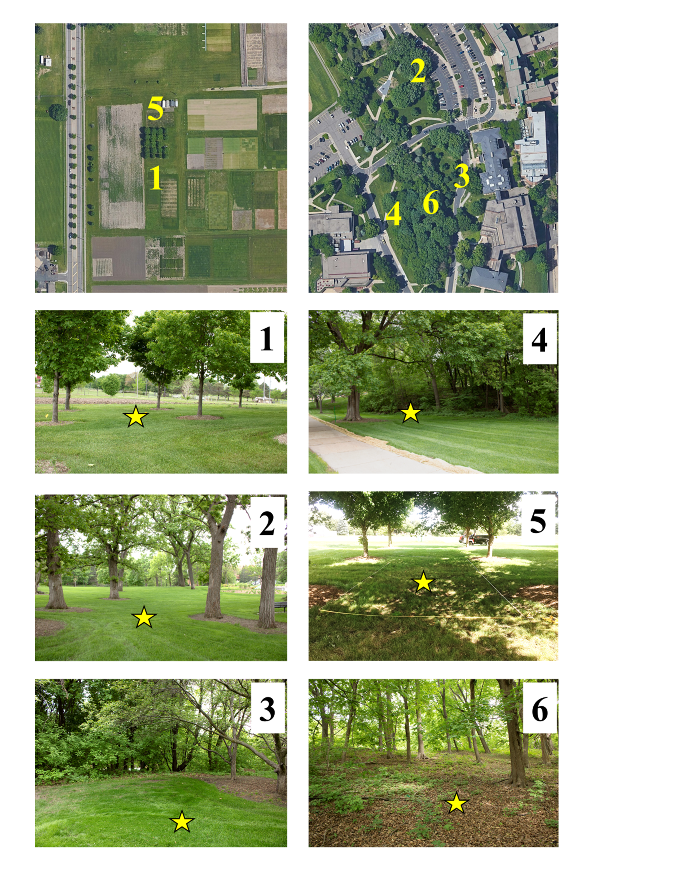
**

**Supplemental Figure S3:** Crop stands in which spectral data collected at the soil surface. A) Wheat field on 30 June 2018 (data were collected on 2 and 6 July 2018). B) Canola on 23 April 2018. C) Barley on 23 April 2018.


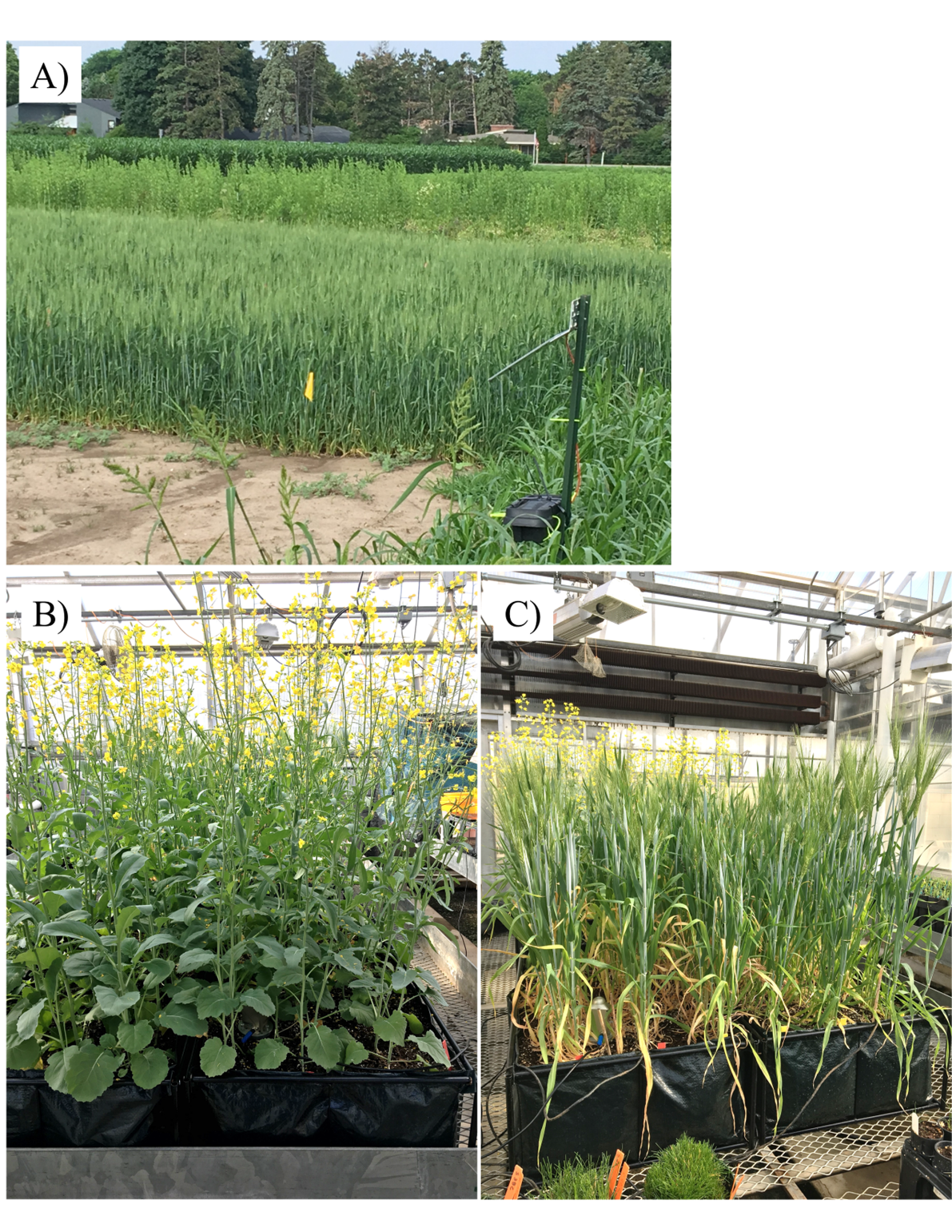


**Supplemental Figure S4:** Results of a linear regression between the narrow- and broadband R:FR ratio for data collected under full sun and under foliar shade in the field. Data within the light red shading fall within a 95% confidence interval for value prediction.


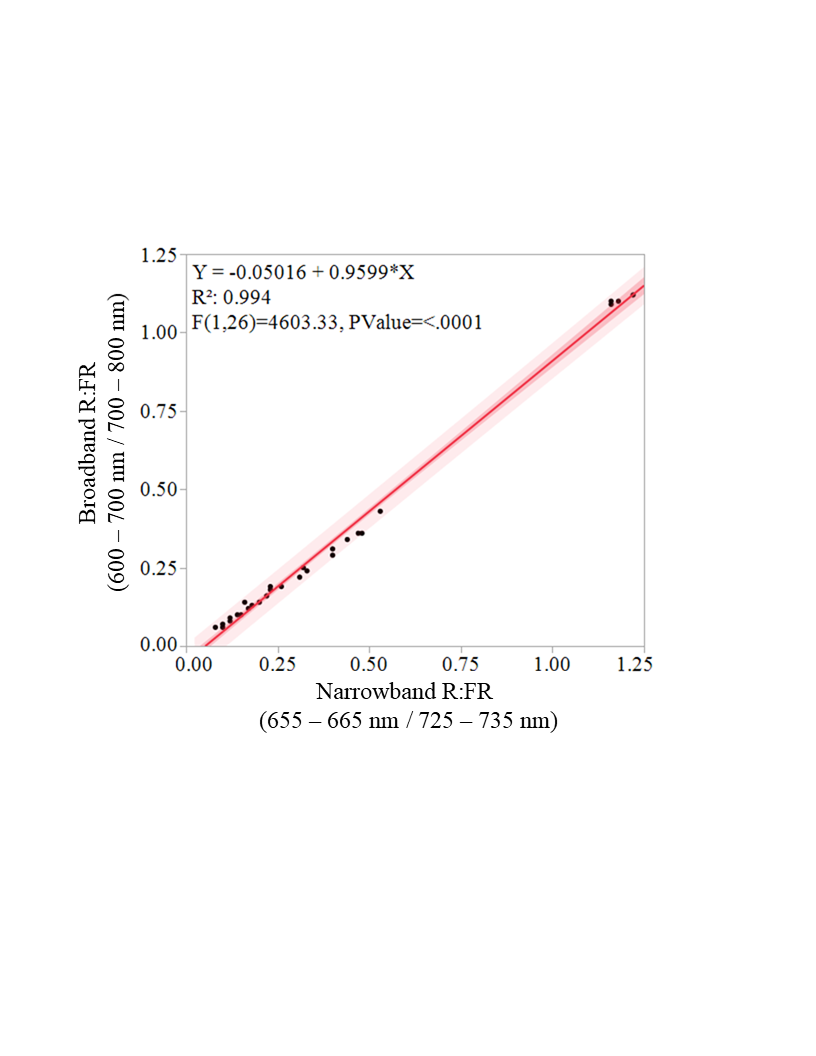


**Supplemental Figure S5:** Results of a linear regression between the narrow- and broadband B:G ratios for A) data collected under full sun and under foliar shade in the field, and B) data collected under foliar shade sites only. The red arrow indicates data collected under full sun. Data within the light blue shading fall within a 95% confidence interval for value prediction.


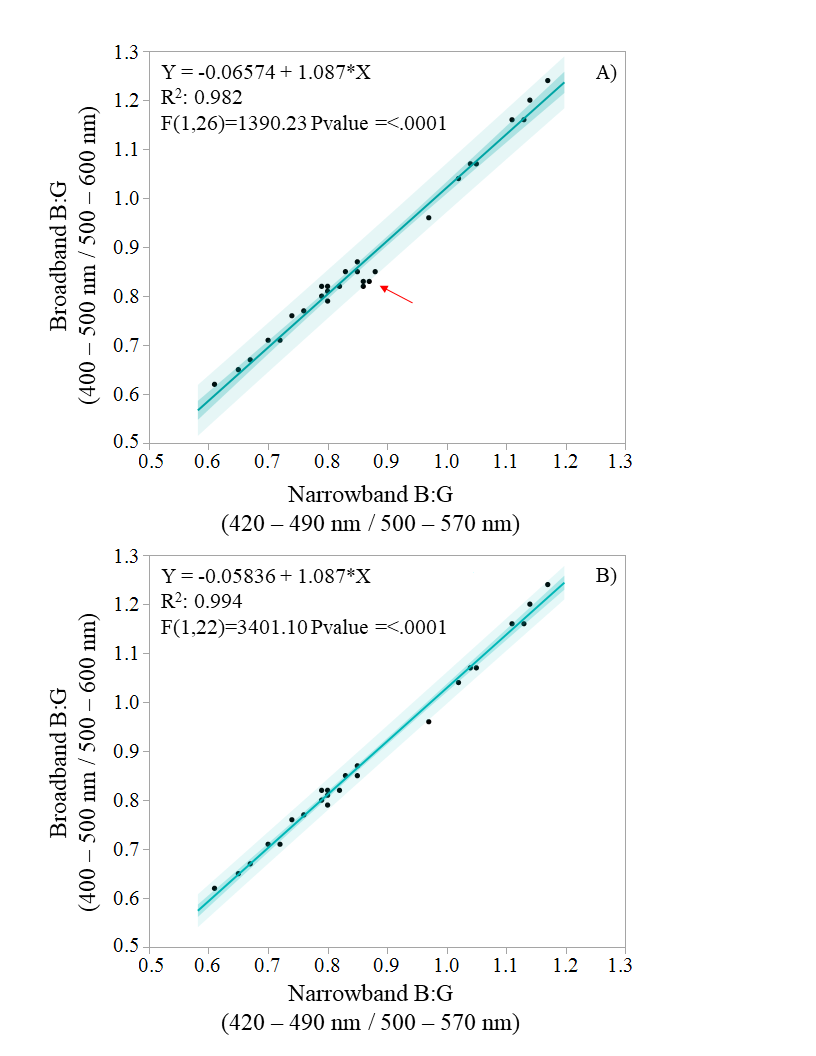


**Supplemental Figure S6:** Results of a linear regression between the narrow- and broadband A) R:FR ratios and B) B:G ratios for all data collectced under single and layered photoselective filters under natural light. The red arrows indicate data collected under Rosco e-colour+ ½ and Full CTB filters and LEE Filters full CTB filters. Data within the light red or blue shading fall within a 95% confidence interval for value prediction.


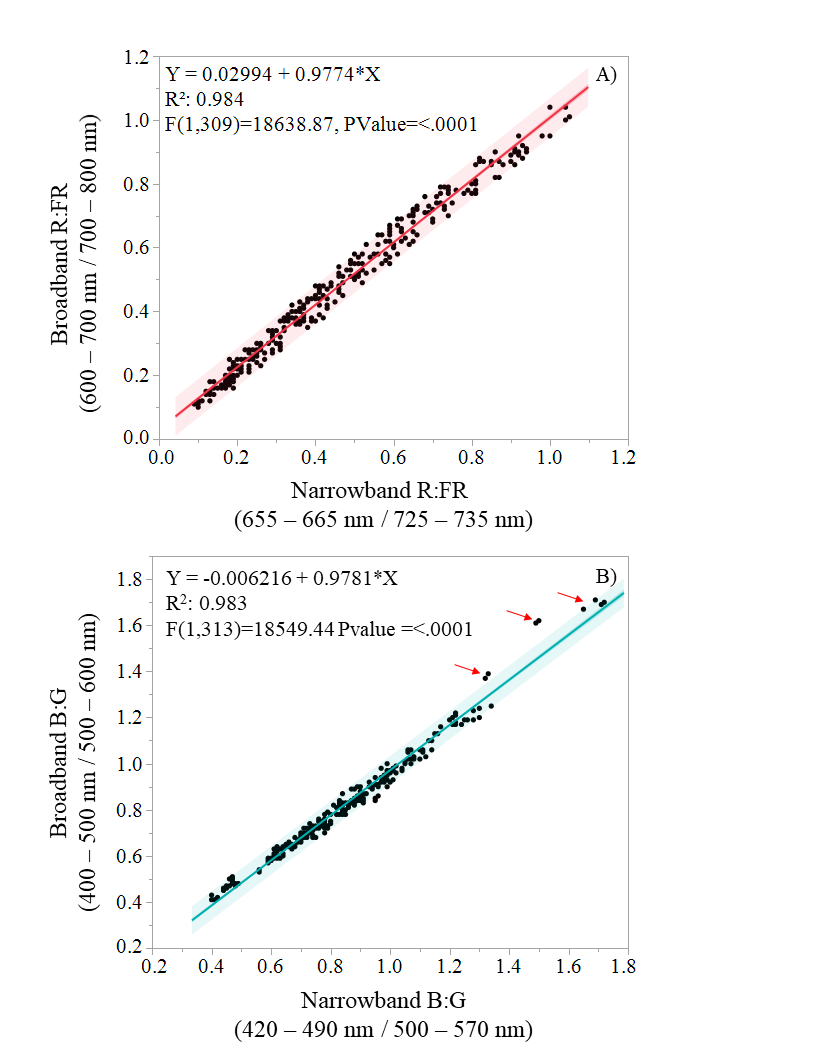


**Supplemental Figure S7:** Results of a linear regression between the narrow- and broadband A) R:FR ratios and B) B:G ratios for all data collectced under single and layered photoselective filters in the greenhouse under supplemental lighting. The red arrows indicates data collected under ½ and LEE Filters and Rosco e-colour+ Full CTB filters under HPS lamps Data within the light red or blue shading fall within a 95% confidence interval for value prediction.


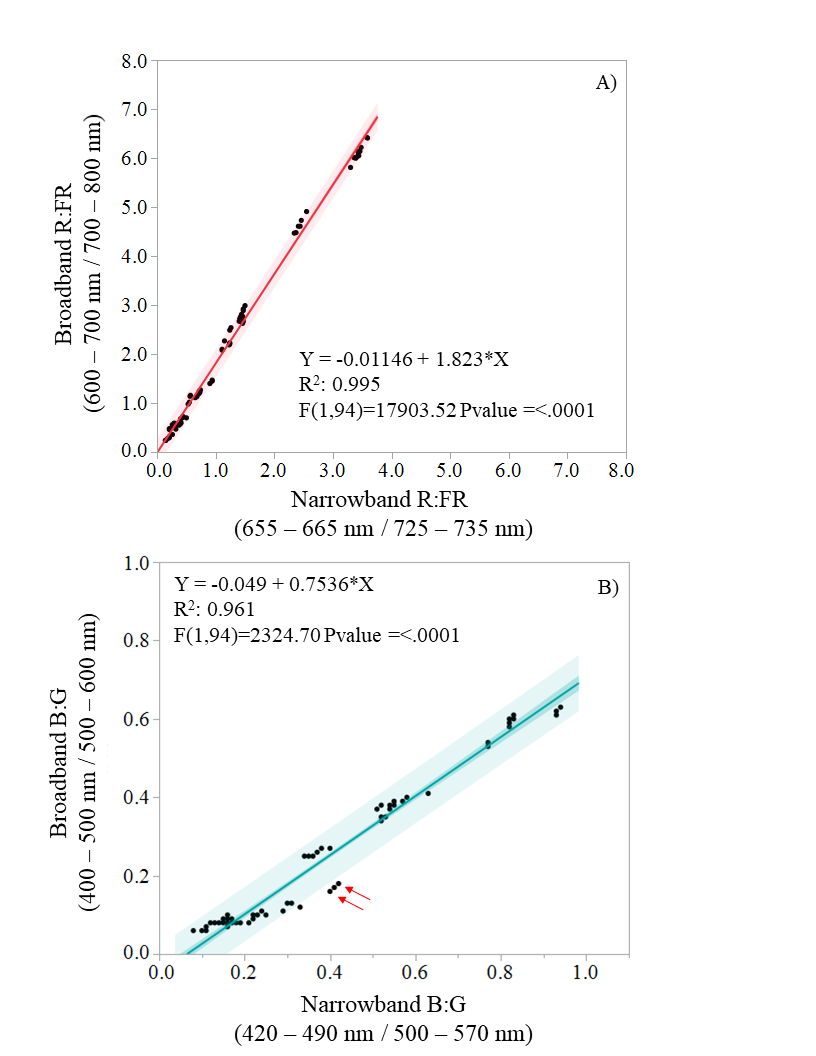


**Supplemental Figure S8:** Relative spectral photon distributions (SPD) acquired under the combination of ⅛ – ½ LEE Filters color temperature blue (CTB) and LEE Filters 0.15 – 0.60 neutral density (ND) filters. A-C) 0.15 ND + ⅛, ¼, or ½ CTB. D-F) 0.30 ND + ⅛, ¼, or ½ CTB. G-I) 0.60 ND + ⅛, ¼, or ½ CTB. Data were normalized to the photon flux at 800 nm and are presented as the average of relative SPDs acquired on 27 May, 30 May, and 12 June 2020 on clear sky or mostly sunny days between 13:00-14:00 h. Data collected under full sun were on average; R:FR = 1.15, B:G = 0.87, % Blue = 29%, % Green = 35%, and % Red = 36%. Red bars indicate 400 and 700 nm respectively, designating photosynthetically active radiation (PAR) between the red bars. Black lines represent the relative SPD of the layered filters, and blue lines represent the SPD of the original CTB filter.


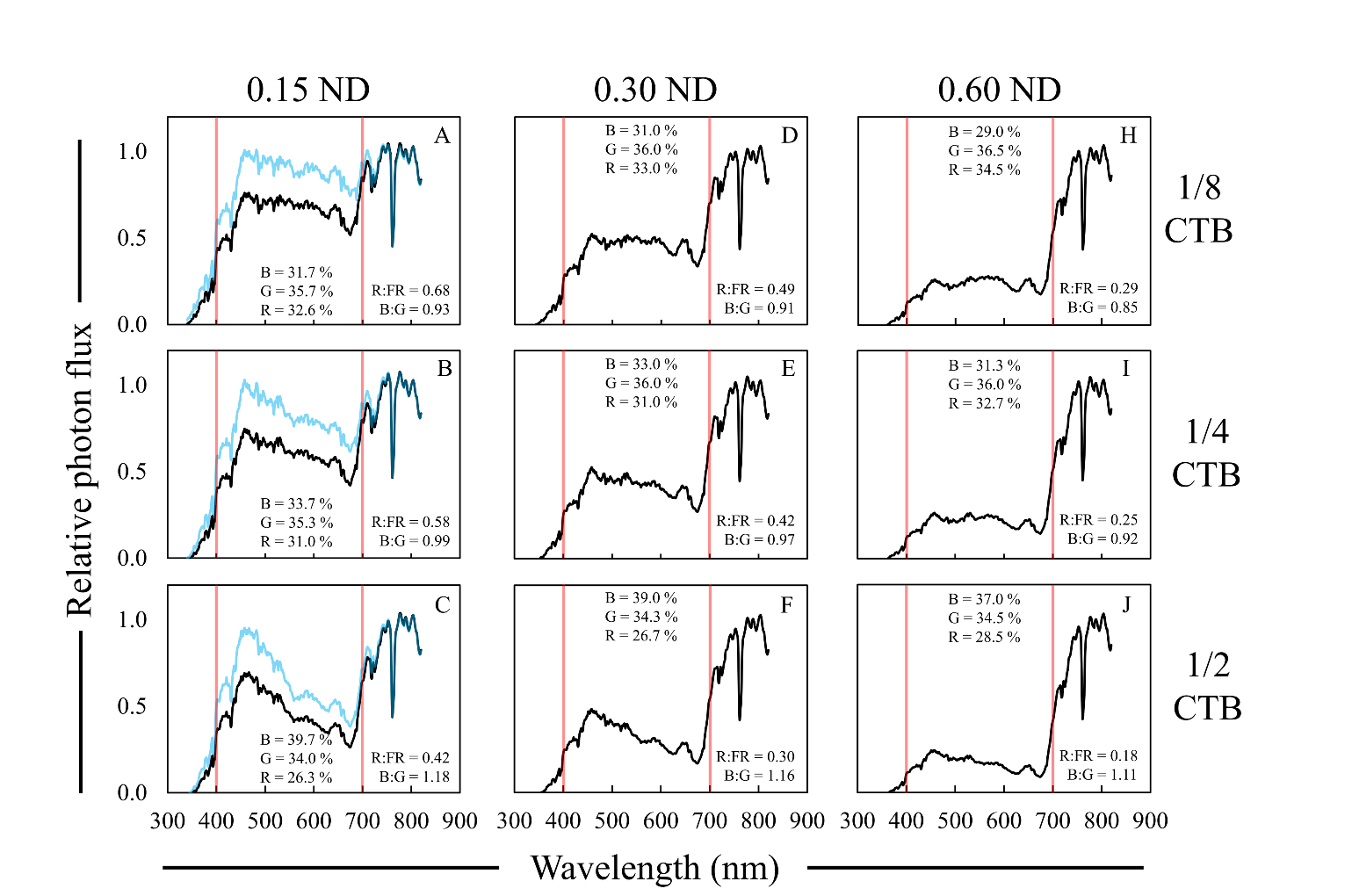


**Supplemental Figure S9:** Relative spectral photon distributions (SPD) acquired under the combination of ¼, ½, or full strength LEE Filters plus green (PG) and LEE Filters 0.15 – 0.60 neutral density (ND) filters. A-C) 0.15 ND + ¼, ½, or full strength PG. D-F) 0.30 ND + ¼, ½, or full strength PG. G-I) 0.60 ND + ¼, ½, or full strength PG. Data were normalized to the photon flux at 800 nm, and are presented as the average of relative SPDs acquired on 27 May, 30 May, and 12 June 2020 on clear sky or mostly sunny days between 13:00-14:00 h. Data collected under full sun were on average; R:FR = 1.15, B:G = 0.87, % Blue = 29%, % Green = 35%, and % Red = 36%. Red bars indicate 400 and 700 nm respectively, designating photosynthetically active radiation (PAR) between the red bars. Black lines represent the relative SPD of the layered filters, and green lines represent the SPD of the original PG filter.


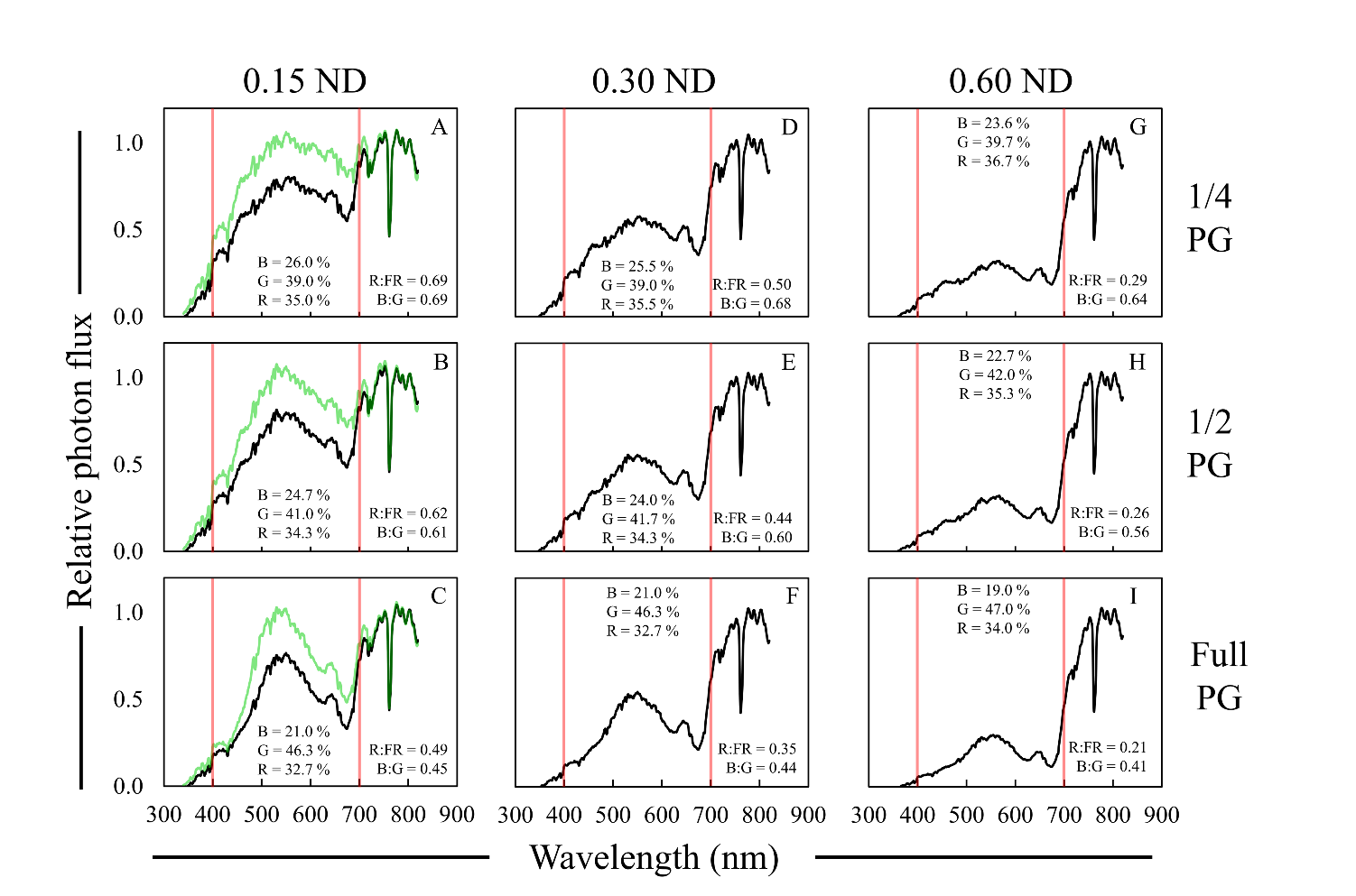


**Supplemental Figure S10:** Relative spectral photon distributions (SPD) acquired under the combination of 1) ⅛, ¼, or ½ LEE Filters color temperature blue (CTB), 2) ¼, ½ , or full strength LEE Filters plus green (PG), and 3) LEE Filters 0.30 or 0.60 neutral density (ND) filters. A-F) Combinations of CTB filters only. G-L) 0.30 ND filter layered on CTB + PG filters. M-R) 0.60 ND filter layered on CTB + PG filters. Data were normalized to the photon flux at 800 nm and are presented as the average of relative SPDs acquired on 27 May, 30 May, and 12 June 2020 on clear sky or mostly sunny days between 13:00-14:00 h. Data collected under full sun were on average; R:FR = 1.15, B:G = 0.87, % Blue = 29%, % Green = 35%, and % Red = 36%. Red bars indicate 400 and 700 nm respectively, designating photosynthetically active radiation (PAR) between the red bars. Black lines represent the relative SPD of the layered filters, blue lines represent the SPD of the original CTB filer (A-F), green lines represent the SPD of the original PG filter (A-F), and teal lines represent the SPD of layered CTB and PG filters (G-R).


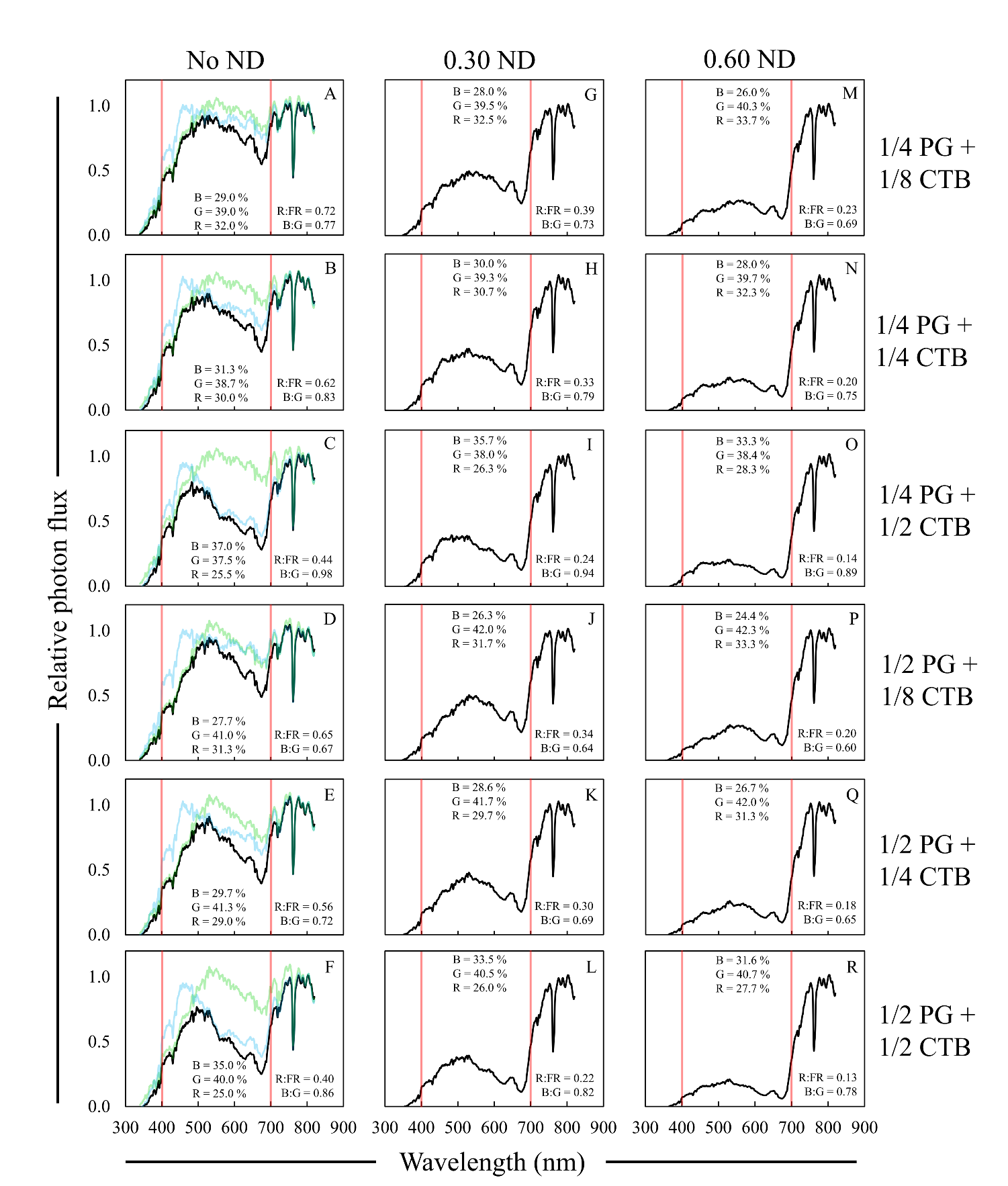


**Supplemental Figure S11:** Two general models for simulating different types of foliar shade. In all panels, black lines in SPDs represent either moderate or deep foliar shade, and colored lines represent the SPD from the designated filter(s). Red bars indicate 400 and 700 nm respectively, designating photosynthetically active radiation (PAR) between the red bars. A) An area of moderate foliar shade with more diffuse light has an SPD with a (1) relatively high B:G ratio and a reduced R:FR ratio. A LEE Filters ¼ CTB filter (2) simulates the B:G ratio of the SPD from the moderate foliar shade site, no other parameters are simulated well. A single LEE Filters 0.30 ND filter (3) similarly does not accurately simulate the moderate foliar shade SPD. The combination of the ¼ CTB and the 0.30 ND filters (4) more accurately simulates the entire moderate foliar shade SPD. B) An area of deep foliar shade with a (1) relatively low B:G ratio and a much lower R:FR ratio. A single LEE Filters ½ PG filter (2) does not simulate the deep shade foliar shade SPD. The combination of a LEE Filters ½ PG and a LEE Filters ¼ CTB filters (3) still does not provide an accurate deep foliar shade simulate. The combination of the PG, CTB, and a LEE Filters 0.60 ND filter (4) more accurately simulates the entire moderate deep shade SPD.


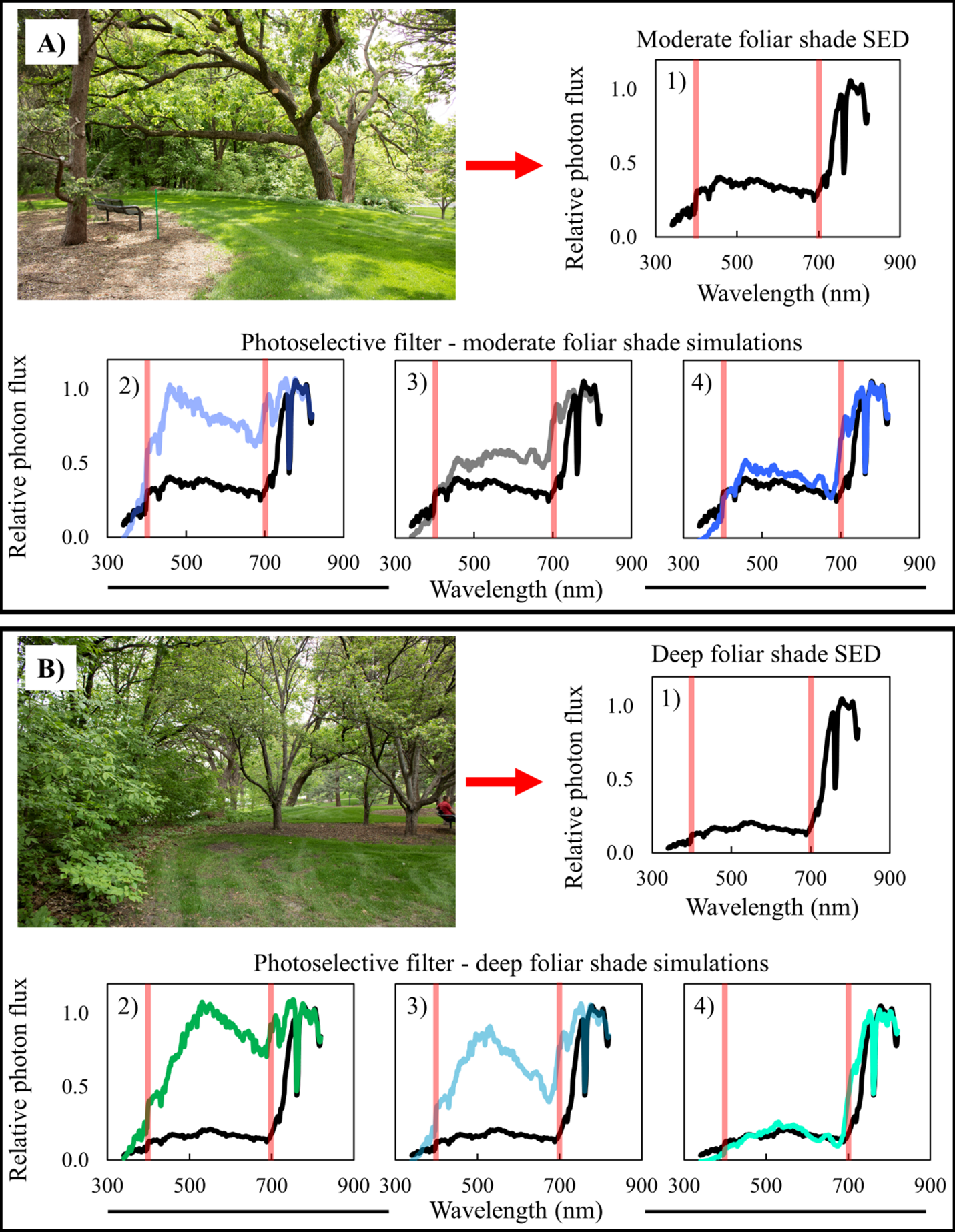

Supplement: Supplementary file 1 — Additional file 1: Figure S1. Spectroradiometer setup to collect spectral data under photoselective filters. A box with a ~ 3.0 x 3.0 cm hole was placed over the spectroradiometer in order to only expose the sensor to sunlight passed through a given photoselective filter. Figure S2. Foliar shade sites at the University of Minnesota St. Paul campus where SPD data were collected in 2018 (1-4, and 6) and in 2020 (5). 1) Maple grove-southern row, 2) Oak grove, 3) Northern forest edge, 4) Southern forest edge, 5) Maple grove-northern row, and 6) Within a forest. Satellite images were acquired through Google Earth at 1250 ft. Pictures of shade sites were adjusted for brightness and contrast to make foliage more visible. Stars indicate the approximate position data were acquired. Figure S3. Crop stands in which spectral data collected at the soil surface. A) Wheat field on 30 June 2018 (data were collected on 2 and 6 July 2018). B) Canola on 23 April 2018. C) Barley on 23 April 2018. Figure S4. Results of a linear regression between the narrow- and broadband R:FR ratio for data collected under full sun and under foliar shade in the field. Data within the light red shading fall within a 95% confidence interval for value prediction. Figure S5. Results of a linear regression between the narrow- and broadband B:G ratios for A) data collected under full sun and under foliar shade in the field, and B) data collected under foliar shade sites only. The red arrow indicates data collected under full sun. Data within the light blue shading fall within a 95% confidence interval for value prediction. Figure S6. Results of a linear regression between the narrow- and broadband A) R:FR ratios and B) B:G ratios for all data collectced under single and layered photoselective filters under natural light. The red arrows indicate data collected under Rosco e-colour+ 1/2 and Full CTB filters and LEE Filters full CTB filters. Data within the light red or blue shading fall within a 95% confidence [file 13007_2022_844_MOESM1_ESM.docx]
