# Supplementary material for: Layering contrasting photoselective filters improves the simulation of foliar shade
Source: Plant Methods. 2022 Feb 8;18:16. doi: 10.1186/s13007-022-00844-8 (PMC8822638; doi:10.1186/s13007-022-00844-8)
Supplement: Supplementary file 2 — Additional file 2: Table S1: Average relative blue, green and red PPF data collected under either full sun or foliar shade. Table S2: Average relative blue, green and red PPF data collected under neutral density (ND) photoselective filters. Table S3: Average relative blue, green and red PPF data collected under color temperature blue (CTB) photoselective filters. Table S4: Average relative blue, green and red PPF data collected under plus green (PG) photoselective filters. Table S5: Average relative blue, green and red PPF data collected under layered Rosco color temperature blue (CTB) neutral density (ND) filters. Table S6: Average relative blue, green and red PPF data collected under layered Rosco plus green (PG) and neutral density (ND) filters. Table S7: Average relative blue, green and red PPF data collected under layered Rosco color temperature blue (CTB), plus green (PG), and neutral density (ND) filters. Table S8: Average data collected under layered LEE Filters color temperature blue (CTB) and neutral density (ND) filters. Table S9: Average data collected under layered LEE Filters plus green (PG) and neutral density (ND) filters. Table S10: Average data collected under layered LEE Filters color temperature blue (CTB), plus green (PG), and neutral density (ND) filters. Table S11: The effects of high-pressure sodium (HPS) lamps on LEE Filters spectral quality. Table S12: The effects of metal halide (MH) lamps on LEE Filters spectral quality. [file 13007_2022_844_MOESM2_ESM.docx]

**Supplemental Table S1:** Average relative blue, green and red PPF data collected under either full sun or foliar shade.

| Site | Blue ^a^ | Green ^b^ | Red ^c^ |
| --- | --- | --- | --- |
|  | % | | |
| Full sun ^d^ | 29.0 ± 0.0 ^e^ | 35.0 ± 0.0 | 36.0 ± 0.6 |
| Maple grove-southern row | 40.8 ± 2.6 | 35.0 ± 0.0 | 24.2 ± 2.6 |
| Oak grove | 37.4 ± 2.5 | 34.8 ± 0.5 | 27.8 ± 2.5 |
| Northern forest edge | 31.0 ± 2.9 | 39.0 ± 0.5 | 30.0 ± 3.3 |
| Southern forest edge | 32.2 ± 1.7 | 38.5 ± 0.6 | 29.3 ± 2.1 |
| Maple grove-northern row | 32.3 ± 1.5 | 40.7 ± 0.5 | 27.0 ± 1.8 |
| Within a forest | 27.7 ± 1.5 | 41.7 ± 1.3 | 30.6 ± 1.7 |
| Under wheat | 28.4 ± 1.7 | 38.6 ± 1.6 | 33.0 ± 1.5 |
| Under barley | 25.6 ± 0.7 | 37.8 ± 0.7 | 36.6 ± 0.4 |
| Under canola | 22.3 ± 1.7 | 43.9 ± 2.3 | 33.8 ± 0.7 |

^a^ Blue = Percentage of PPF between 400-499 nm relative to total PPF

^b^ Green = Percentage of PPF between 500-599 nm relative to total PPF

^c^ Red = Percentage of PPF between 600-700 nm relative to total PPF

^d^ Site description and site number in parentheses

^e^ Mean ± standard deviation; Data are presented as the average from the following dates for each site: 28 May, 13 June, 2 July, and 6 July for 2018 (full sun, sites 1-4, and site 6), 30 May, 31 May, 12 June, and 11 Aug. 2020 (site 5), 2 and 6 July 2018 (wheat), and 23 April 2018 (barley and canola). Data were acquired between 13:00-14:00 h on clear sky or mostly sunny days only.

**Supplemental Table S2:** Average relative blue, green and red PPF data collected under neutral density (ND) photoselective filters.

| Filter | Brand | Blue ^a^ | Green ^b^ | Red ^c^ |
| --- | --- | --- | --- | --- |
|  |  | % | | |
| 0.15 ND | LEE | 28.0 E ^d^ | 35.5 CD | 36.5 C |
|  | e-colour+ | 29.0 D | 36.0 B | 35.0 D |
|  | Cinegel | 29.0 D | 35.0 D | 36.0 C |
| 0.30 ND | LEE | 28.0 E | 36.0 B | 36.0 C |
|  | e-colour+ | 30.0 C | 36.0 B | 34.0 F |
|  | Cinegel | 29.3 D | 35.7 BC | 35.0 D |
| 0.60 ND | LEE | 26.0 F | 36.3 B | 37.7 B |
|  | e-colour+ | 31.0 B | 37.0 A | 32.0 G |
|  | Cinegel | 30.3 C | 35.0 D | 34.7 DE |
| 0.90 ND | LEE | 25.0 G | 36.0 B | 39.0 A |
|  | e-colour+ | 32.0 A | 37.0 A | 31.0 H |
|  | Cinegel | 31.0 B | 35.0 D | 34.0 EF |

^a^ Blue = Percentage of PPF between 400-499 nm relative to total PPF

^b^ Green = Percentage of PPF between 500-599 nm relative to total PPF

^c^ Red = Percentage of PPF between 600-700 nm relative to total PPF

^d^ Data are presented as averages acquired on three different clear sky or mostly sunny days between 13:00-14:00 h: 27 May, 30 May, and 12 June 2020. Means are only compared within column and were separated with Fisher’s LSD. Means followed by a common letter are not significantly different (*P* = 0.05). Data collected under full sun were on average; % Blue = 29%, % Green = 35%, % Red = 36%, and PPF = 1719 µmol m^-2^ s^-1^.

**Supplemental Table S3:** Average relative blue, green and red PPF data collected under color temperature blue (CTB) photoselective filters.

| Filter | Brand | Blue ^a^ | Green ^b^ | Red ^c^ |
| --- | --- | --- | --- | --- |
|  |  | % | | |
| 1/8 CTB | LEE | 32.0 IJ ^d^ | 35.0 A | 33.0 B |
|  | e-colour+ | 32.3 I | 33.7 B | 33.0 B |
|  | Cinegel | 31.0 J | 35.0 A | 34.0 A |
| 1/4 CTB | LEE | 34.0 H | 35.0 A | 31.0 C |
|  | e-colour+ | 35.0 G | 33.7 B | 31.0 C |
|  | Cinegel | 34.7 GH | 34.3 B | 31.0 C |
| 1/2 CTB | LEE | 40.0 E | 34.0 B | 26.0 F |
|  | e-colour+ | 42.0 D | 30.0 D | 28.0 D |
|  | Cinegel | 39.4 F | 33.3 C | 27.3 E |
| Full CTB | LEE | 49.3 B | 29.0 E | 21.7 H |
|  | e-colour+ | 47.3 C | 29.3 E | 23.4 H |
|  | Cinegel | 51.0 A | 30.0 D | 19.0 I |

^a^ Blue = Percentage of PPF between 400-499 nm relative to total PPF

^b^ Green = Percentage of PPF between 500-599 nm relative to total PPF

^c^ Red = Percentage of PPF between 600-700 nm relative to total PPF

^d^ Data are presented as averages acquired on three different clear sky or mostly sunny days between 13:00-14:00 h: 27 May, 30 May, and 12 June 2020. Means are only compared within column and were separated with Fisher’s LSD. Means followed by a common letter are not significantly different (*P* = 0.05). Data collected under full sun were on average; % Blue = 29%, % Green = 35%, % Red = 36%, and PPF = 1719 µmol m^-2^ s^-1^.

**Supplemental Table S4:** Average relative blue, green and red PPF data collected under plus green (PG) photoselective filters.

| Filter | Brand | Blue ^a^ | Green ^b^ | Red ^c^ |
| --- | --- | --- | --- | --- |
|  |  | % | | |
| 1/4 PG | LEE | 26.3 AB ^d^ | 38.7 DE | 35.0 ABC |
|  | e-colour+ | 25.3 CD | 39.0 DE | 35.7 AB |
|  | Cinegel | 27.0 A | 38.0 E | 35.0 A |
| 1/2 PG | LEE | 25.0 CD | 41.0 C | 34.0 C |
|  | e-colour+ | 25.0 D | 40.0 CD | 35.0 BC |
|  | Cinegel | 26.0 BC | 41.0 C | 33.0 DE |
| Full  PG | LEE | 21.0 F | 46.7A | 32.3 DE |
|  | e-colour+ | 21.3 F | 45.7 B | 33.0 D |
|  | Cinegel | 22.7 E | 46.0 AB | 31.3 E |

^a^ Blue = Percentage of PPF between 400-499 nm relative to total PPF

^b^ Green = Percentage of PPF between 500-599 nm relative to total PPF

^c^ Red = Percentage of PPF between 600-700 nm relative to total PPF

^d^ Data are presented as averages acquired on three different clear sky or mostly sunny days between 13:00-14:00 h: 27 May, 30 May, and 12 June 2020. Means are only compared within column and were separated with Fisher’s LSD. Means followed by a common letter are not significantly different (*P* = 0.05). Data collected under full sun were on average; % Blue = 29%, % Green = 35%, % Red = 36%, and PPF = 1719 µmol m^-2^ s^-1^

**Supplemental Table S5:** Average relative blue, green and red PPF data collected under layered Rosco color temperature blue (CTB) neutral density (ND) filters.

| Filter(s) | Blue ^a^ | Green ^b^ | Red ^c^ |
| --- | --- | --- | --- |
|  | % | | |
| 0.15 ND ^d^ | 29.0 K ^e^ | 36.0 B | 35.0 A |
| 0.30 ND | 30.0 J | 36.0 B | 34.0 B |
| 0.60 ND | 31.0 I | 37.0 A | 32.0 C |
| 1/8 CTB | 31.0 I | 35.0 C | 34.0 B |
| 1/4 CTB | 34.7 F | 34.3 EF | 31.0 D |
| 1/2 CTB | 39.4 C | 33.3 G | 27.3 G |
| 1/8 CTB |  |  |  |
| +0.15 ND | 32.3 H | 35.7 B | 32.0 C |
| +0.30 ND | 32.7 H | 36.0 B | 31.0 D |
| +0.60 ND | 33.7 G | 36.7 A | 29.6 E |
| 1/4 CTB |  |  |  |
| +0.15 ND | 35.6 F | 34.7 CD | 29.7 E |
| +0.30 ND | 36.3 E | 35.0 C | 28.4 F |
| +0.60 ND | 37.3 D | 35.7 B | 27.0 G |
| 1/2 CTB |  |  |  |
| +0.15 ND | 40.6 B | 33.7 F | 25.7 H |
| +0.30 ND | 41.0 AB | 34.0 EF | 25.0 I |
| +0.60 ND | 41.7 A | 34.3 DE | 24.0 J |

^a^ Blue = Percentage of PPF between 400-499 nm relative to total PPF

^b^ Green = Percentage of PPF between 500-599 nm relative to total PPF

^c^ Red = Percentage of PPF between 600-700 nm relative to total PPF

^d^ Rosco Cinegel was used for CTB and Rosco e-colour+ was used for ND filters

^e^ Data are presented as averages acquired on three different clear sky or mostly sunny days between 13:00-14:00 h: 27 May, 30 May, and 12 June 2020. Means are only compared within column and were separated with Fisher’s LSD. Means followed by a common letter are not significantly different (*P* = 0.05). Data collected under full sun were on average; % Blue = 29%, % Green = 35%, % Red = 36%, and PPF = 1719 µmol m^-2^ s^-1^

**Supplemental Table S6:** Average relative blue, green and red PPF data collected under layered Rosco plus green (PG) and neutral density (ND) filters.

| Filter(s) | Blue ^a^ | Green ^b^ | Red ^c^ |
| --- | --- | --- | --- |
|  | % | | |
| 0.15 ND ^d^ | 29.0 C ^e^ | 36.0 G | 35.0 AB |
| 0.30 ND | 30.0 B | 36.0 G | 34.0 CD |
| 0.60 ND | 31.0 A | 37.0 G | 32.0 G |
| 1/4 PG | 25.3 GHI | 39.0 F | 35.7 A |
| 1/2 PG | 25.0 I | 40.0 EF | 35.0 BC |
| Full PG | 21.3 K | 45.7 B | 33.0 ED |
| 1/4 PG |  |  |  |
| +0.15 ND | 26.6 EF | 39.0 F | 34.4 CD |
| +0.30 ND | 27.0 E | 40.0 EF | 33.0 DE |
| +0.60 ND | 27.7 D | 40.7 E | 31.6 G |
| 1/2 PG |  |  |  |
| +0.15 ND | 25.4 FGH | 42.3 D | 32.3 FG |
| +0.30 ND | 25.0 HI | 42.7 CD | 32.3 G |
| +0.60 ND | 25.7 FG | 43.3 C | 31.0 H |
| Full PG |  |  |  |
| +0.15 ND | 22.0 JK | 46.0 A | 32.0 G |
| +0.30 ND | 22.0 JK | 47.0 A | 31.0 H |
| +0.60 ND | 22.7 J | 47.3 A | 30.0 I |

^a^ Blue = Percentage of PPF between 400-499 nm relative to total PPF

^b^ Green = Percentage of PPF between 500-599 nm relative to total PPF

^c^ Red = Percentage of PPF between 600-700 nm relative to total PPF

^d^ Rosco Cinegel was used for CTB and Rosco e-colour+ was used for ND filters

^e^ Data are presented as averages acquired on three different clear sky or mostly sunny days between 13:00-14:00 h: 27 May, 30 May, and 12 June 2020. Means are only compared within column and were separated with Fisher’s LSD. Means followed by a common letter are not significantly different (*P* = 0.05). Data collected under full sun were on average; % Blue = 29%, % Green = 35%, % Red = 36%, and PPF = 1719 µmol m^-2^ s^-1^

**Supplemental Table S7:** Average relative blue, green and red PPF data collected under layered Rosco color temperature blue (CTB), plus green (PG), and neutral density (ND) filters.

| Filter(s) | Blue ^a^ | Green ^b^ | Red ^c^ |
| --- | --- | --- | --- |
|  | % | | |
| 1/4 PG+ ^d^  1/8 CTB | 28.3 L ^e^ | 38.4 H | 33.3 A |
| +0.30 ND | 29.0 K | 39.7 F | 31.3 C |
| +0.60 ND | 30.0 J | 40.0 EF | 29.7 D |
|  |  |  |  |
| 1/4 PG+  1/4 CTB | 31.3 H | 38.0 H | 30.7 C |
| +0.30 ND | 32.0 G | 39.3 G | 28.7 E |
| +0.60 ND | 32.7 F | 40.0 EF | 27.3 G |
|  |  |  |  |
| 1/4 PG+  1/2 CTB | 35.7 C | 37.3 I | 27.0 G |
| +0.30 ND | 36.3 B | 38.0 H | 25.7 I |
| +0.60 ND | 37.4 A | 38.3 H | 24.3 K |
|  |  |  |  |
| 1/2 PG+  1/8 CTB | 26.3 O | 41.7 C | 32.0 B |
| +0.30 ND | 27.3 N | 42.7 B | 30.0 D |
| +0.60 ND | 27.7 M | 43.3 A | 29.0 E |
|  |  |  |  |
| 1/2 PG+  1/4 CTB | 29.3K | 41.0 D | 29.7 D |
| +0.30 ND | 30.0 J | 42.0 C | 28.0 F |
| +0.60 ND | 30.5 I | 42.5 B | 27.0 G |
|  |  |  |  |
| 1/2 PG+  1/2 CTB | 33.0 F | 40.5 E | 26.5 H |
| +0.30 ND | 34.0 E | 41.0 D | 25.0 J |
| +0.60 ND | 34.5 D | 41.5 C | 24.0 K |

^a^ Blue = Percentage of PPF between 400-499 nm relative to total PPF

^b^ Green = Percentage of PPF between 500-599 nm relative to total PPF

^c^ Red = Percentage of PPF between 600-700 nm relative to total PPF

^d^ Rosco Cinegel was used for CTB and Rosco e-colour+ was used for ND filters

^e^ Data are presented as averages acquired on three different clear sky or mostly sunny days between 13:00-14:00 h: 27 May, 30 May, and 12 June 2020. Means are only compared within column and were separated with Fisher’s LSD. Means followed by a common letter are not significantly different (*P* = 0.05). Data collected under full sun were on average; % Blue = 29%, % Green = 35%, % Red = 36%, and PPF = 1719 µmol m^-2^ s^-1^

**Supplemental Table S8:** Average data collected under layered LEE Filters color temperature blue (CTB) and neutral density (ND) filters.

| Filter(s) | R:FR ^a^ | PPE ^b^ | B:G ^c^ | Blue ^d^ | Green ^e^ | Red ^f^ | PPF reduction ^g^ | UV-A PF ^h^ |
| --- | --- | --- | --- | --- | --- | --- | --- | --- |
|  |  |  |  | % | | | | µmol m^-2^ s^-1^ |
| 0.15 ND | 0.85 B ^i^ | 0.69 A | 0.84 H | 28.0 G | 35.5 CD | 36.5 B | 29.7 H | 50.4 AB |
| 0.30 ND | 0.61 E | 0.64 D | 0.83 H | 28.0 G | 36.0 B | 36.0 B | 51.7 F | 27.3 DE |
| 0.60 ND | 0.36 I | 0.56 H | 0.78 I | 26.0 H | 36.3 B | 37.7A | 76.0 C | 10.1 HI |
| 1/8 CTB | 0.91 A | 0.69 A | 0.94 F | 32.0 EF | 35.0 DE | 33.0 D | 20.7 I | 58.5 A |
| 1/4 CTB | 0.78 C | 0.67 B | 1.01 D | 34.0 D | 35.0 DE | 31.0 E | 28.3 H | 48.5 B |
| 1/2 CTB | 0.57 F | 0.63 E | 1.21 A | 40.0 A | 34.0 G | 26.0 G | 44.0 G | 38.2 C |
| 1/8 CTB |  |  |  |  |  |  |  |  |
| +0.15 ND | 0.68 D | 0.66 C | 0.93 FG | 31.7 F | 35.7 BC | 32.6 D | 46.3 G | 27.7 D |
| +0.30 ND | 0.49 G | 0.61 F | 0.91 G | 31.0 F | 36.0 B | 33.0 D | 58.3 E | 19.2 EFG |
| +0.60 ND | 0.29 J | 0.52 J | 0.85 H | 29.0 G | 36.5 A | 34.5 C | 81.7 B | 6.3 I |
| 1/4 CTB |  |  |  |  |  |  |  |  |
| +0.15 ND | 0.58 EF | 0.64 D | 0.99 DE | 33.7 D | 35.3 DE | 31.0 E | 52.0 F | 26.6 DE |
| +0.30 ND | 0.42 H | 0.59 G | 0.97 E | 33.0 DE | 36.0 B | 31.0 E | 66.0 D | 16.3 FGH |
| +0.60 ND | 0.25 K | 0.49 K | 0.92 FG | 31.3 F | 36.0 B | 32.7 D | 82.0 B | 2.9 I |
|  |  |  |  |  |  |  |  |  |
| 1/2 CTB |  |  |  |  |  |  |  |  |
| +0.15 ND | 0.42 H | 0.59 G | 1.18 B | 39.7 AB | 34.0 G | 26.3 G | 61.3 E | 21.9 DEF |
| +0.30 ND | 0.30 J | 0.54 I | 1.16 B | 39.0 B | 34.3 FG | 26.7 G | 74.0 C | 13.1 GHI |
| +0.60 ND | 0.18 L | 0.44 L | 1.11 C | 37.0 C | 34.5 EF | 28.5 F | 87.0 A | 4.8 I |

^a^ R:FR = 655-665 / 725-735

^b^ PPE = Phytochrome photoequilibria

^c^ B:G = 420-490 / 500-570

^d^ Blue = Percentage of PPF between 400-499 nm relative to total PPF

^e^ Green = Percentage of PPF between 500-599 nm relative to total PPF

^f^ Red = Percentage of PPF between 600-700 nm relative to total PPF

^g^ PPF reduction = Percent reduction in PPF relative to full sun

^h^ UV-A PF = Photon flux between 340-399 nm

^i^ Data are presented as averages acquired on three different clear sky or mostly sunny days between 13:00-14:00 h: 27 May, 30 May, and 12 June 2020. Means are only compared within column and were separated with Fisher’s LSD. Means followed by a common letter are not significantly different (*P* = 0.05).

**Supplemental Table S9:** Average data collected under layered LEE Filters plus green (PG) and neutral density (ND) filters.

| Filters | R:FR ^a^ | PPE ^b^ | B:G ^c^ | Blue ^d^ | Green ^e^ | Red ^f^ | PPF reduction ^g^ | UV-A PF ^h^ |
| --- | --- | --- | --- | --- | --- | --- | --- | --- |
|  |  |  |  | % | | | | µmol m^-2^ s^-1^ |
| 0.15 ND | 0.85 B ^i^ | 0.69 B | 0.84 A | 28.0 A | 35.5 E | 36.5 B | 29.7 H | 50.44 A |
| 0.30 ND | 0.61 E | 0.64 E | 0.83 A | 28.0 A | 36.0 E | 36.0 BC | 51.7 F | 27.3 B |
| 0.60 ND | 0.36 H | 0.56 J | 0.78 B | 26.0 BC | 36.3 E | 37.7 A | 76.0 C | 10.1 DEFG |
| 1/4 PG | 0.97 A | 0.70A | 0.71 C | 26.3 B | 38.7 D | 35.0 C | 20.0 J | 54.6 A |
| 1/2 PG | 0.86 B | 0.69 B | 0.62 F | 25.0 D | 41.0 C | 34.3 D | 25.7 I | 47.8 A |
| PG | 0.65 D | 0.67 C | 0.44 I | 21.0 H | 46.7 A | 32.3 E | 39.3 G | 30.4 B |
| 1/4 PG |  |  |  |  |  |  |  |  |
| +0.15 ND | 0.69 C | 0.67 C | 0.69 D | 26.0 BC | 39.0 D | 35.0 C | 43.0 G | 27.8 B |
| +0.30 ND | 0.50 F | 0.62 G | 0.68 D | 25.5 CD | 39.0 D | 35.5 C | 60.7 E | 16.0 CD |
| +0.60 ND | 0.29 I | 0.52 K | 0.64 E | 23.6 F | 39.7 D | 36.7 B | 81.7 B | 5.6 EFG |
| 1/2 PG |  |  |  |  |  |  |  |  |
| +0.15 ND | 0.62 DE | 0.66 D | 0.61 FG | 24.7 DE | 41.0 C | 34.3 D | 50.0 F | 22.4 BC |
| +0.30 ND | 0.44 G | 0.60 H | 0.60G | 24.0 EF | 41.7 BC | 34.3 D | 71.3 D | 13.3 DEF |
| +0.60 ND | 0.26 I | 0.51 L | 0.56 H | 22.7 G | 42.0 B | 35.3 C | 83.7 AB | 4.7 FG |
| PG |  |  |  |  |  |  |  |  |
| +0.15 ND | 0.49 F | 0.63 F | 0.45 I | 21.0 H | 46.3 A | 32.7 E | 61.0 E | 13.9 CDE |
| +0.30 ND | 0.35 H | 0.58 I | 0.44 I | 21.0 H | 46.3 A | 32.7 E | 72.7 CD | 8.6 DEFG |
| +0.60 ND | 0.21 J | 0.48 M | 0.41 J | 19.0 I | 47.0 A | 34.0 D | 86.0 A | 2.8 G |

^a^ R:FR = 655-665 / 725-735

^b^ PPE = Phytochrome photoequilibria

^c^ B:G = 420-490 / 500-570

^d^ Blue = Percentage of PPF between 400-499 nm relative to total PPF

^e^ Green = Percentage of PPF between 500-599 nm relative to total PPF

^f^ Red = Percentage of PPF between 600-700 nm relative to total PPF

^g^ PPF reduction = Percent reduction in PPF relative to full sun

^h^ UV-A PF = Photon flux between 340-399 nm

^i^ Data are presented as averages acquired on three different clear sky or mostly sunny days between 13:00-14:00 h: 27 May, 30 May, and 12 June 2020. Means are only compared within column and were separated with Fisher’s LSD. Means followed by a common letter are not significantly different (*P* = 0.05).

**Supplemental Table S10:** Average data collected under layered LEE Filters color temperature blue (CTB), plus green (PG), and neutral density (ND) filters.

| Filters | R:FR ^a^ | PPE ^b^ | B:G ^c^ | Blue ^d^ | Green ^e^ | Red ^f^ | PPF reduction ^g^ | UV-A PF ^h^ |
| --- | --- | --- | --- | --- | --- | --- | --- | --- |
|  |  |  |  | % | | | | µmol m^-2^ s^-1^ |
| 1/4 PG+  1/8 CTB | 0.72 A ^i^ | 0.67 A | 0.77 I | 29.0 I | 39.0 K | 32.0 B | 35.0 Q | 38.9 A |
| +0.30 ND | 0.39 F | 0.59 G | 0.73 K | 28.0 L | 39.5 HI | 32.5 B | 68.3 K | 13.4 F |
| +0.60 ND | 0.23 IJ | 0.49 M | 0.69 M | 26.0 P | 40.3 G | 33.7 A | 84.0 E | 5.2 I |
| 1/4 PG+  1/4 CTB | 0.62 C | 0.65 C | 0.83 E | 31.3 F | 38.7 L | 30.0 E | 41.7 O | 32.1 C |
| +0.30 ND | 0.33 G | 0.56 I | 0.79 G | 30.0 G | 39.3 J | 30.7 D | 71.7 I | 11.7 FG |
| +0.60 ND | 0.20 L | 0.46 O | 0.75 J | 28.0 K | 39.7 I | 32.3 B | 85.0 D | 4.5 I |
| 1/4 PG+  1/2 CTB | 0.44 E | 0.61 E | 0.98 A | 37.0 A | 37.5 N | 25.5 J | 52.3 M | 27.1 D |
| +0.30 ND | 0.24 I | 0.51 K | 0.94 B | 35.7 B | 38.0 M | 26.3 I | 77.0 G | 9.8 GH |
| +0.60 ND | 0.14 N | 0.41 Q | 0.89 C | 33.3 E | 38.4 L | 28.3 G | 88.3 B | 3.8 I |
| 1/2 PG+  1/8 CTB | 0.65 B | 0.66 B | 0.67 N | 27.7 M | 41.0 D | 31.3 C | 39.7 P | 35.1 B |
| +0.30 ND | 0.34 G | 0.57 H | 0.64 P | 26.3 JO | 42.0 B | 31.7 C | 70.3 J | 12.2 FG |
| +0.60 ND | 0.20 KL | 0.47 N | 0.60 Q | 24.4 Q | 42.3 A | 33.3 A | 85.3 D | 4.7 I |
| 1/2 PG+  1/4 CTB | 0.56 D | 0.64 D | 0.72 L | 29.7 H | 41.3 E | 29.0 F | 46.0 N | 28.7 D |
| +0.30 ND | 0.30 H | 0.55 J | 0.69 M | 28.6 J | 41.7 C | 29.7 E | 73.3 H | 10.2 GH |
| +0.60 ND | 0.18 M | 0.45 P | 0.65 O | 26.7 N | 42.0 B | 31.3 C | 86.3 C | 4.0 I |
| 1/2 PG+  1/2 CTB | 0.40 F | 0.60 F | 0.86 D | 35.0 C | 40.0 H | 25.0 J | 55.3 L | 24.3 E |
| +0.30 ND | 0.22 JK | 0.49 L | 0.82 F | 33.5 D | 40.5 F | 26.0 J | 78.7 F | 8.7 H |
| +0.60 ND | 0.13 N | 0.39 R | 0.78 H | 31.6 F | 40.7 E | 27.7 H | 89.3 A | 3.3 I |

^a^ R:FR = 655-665 / 725-735

^b^ PPE = Phytochrome photoequilibria

^c^ B:G = 420-490 / 500-570

^d^ Blue = Percentage of PPF between 400-499 nm relative to total PPF

^e^ Green = Percentage of PPF between 500-599 nm relative to total PPF

^f^ Red = Percentage of PPF between 600-700 nm relative to total PPF

^g^ PPF reduction = Percent reduction in PPF relative to full sun

^h^ UV-A PF = Photon flux between 340-399 nm

^i^ Data are presented as averages acquired on three different clear sky or mostly sunny days between 13:00-14:00 h: 27 May, 30 May, and 12 June 2020. Means are only compared within column and were separated with Fisher’s LSD. Means followed by a common letter are not significantly different (*P* = 0.05).

**Supplemental Table S11:** The effects of high-pressure sodium (HPS) lamps on LEE Filters spectral quality.

| Filter(s) | R:FR ^a^ | PPE ^b^ | B:G ^c^ | Blue ^d^ | Green ^e^ | Red ^f^ | PPF reduction ^g^ |
| --- | --- | --- | --- | --- | --- | --- | --- |
|  |  |  |  | % | | | |
| Lamps only | 3.45 A ^h^ | 0.87 A | 0.23 C | 6.0 C | 59.3 E | 34.7 A | - |
| 0.60 ND | 0.93 D | 0.80 C | 0.17 D | 4.9 D | 62.6 D | 32.5 B | 77.0 D |
| 1/2 CTB | 1.46 C | 0.84 B | 0.42 A | 10.8 A | 59.7 E | 29.5 D | 50.7 E |
| 1/2 PG | 2.38 B | 0.87 A | 0.16 DE | 5.3 D | 62.4 D | 32.3 B | 23.0 F |
|  |  |  |  |  |  |  |  |
| 0.60 ND+  1/2 CTB | 0.42 F | 0.74 E | 0.28 B | 7.9 B | 63.7 C | 28.4 E | 89.3 B |
|  |  |  |  |  |  |  |  |
| 0.60 ND+  1/2 PG | 0.67 E | 0.79 D | 0.10 F | 4.0 E | 66.0 B | 30.0 C | 82.3 C |
|  |  |  |  |  |  |  |  |
| 0.60 ND+  1/2 PG+  1/2 CTB | 0.3 G | 0.71 F | 0.14 E | 6.0 C | 67.3 A | 26.7 F | 91.3 A |

^a^ R:FR = 655-665 / 725-735

^b^ PPE = Phytochrome photoequilibria

^c^ B:G = 420-490 / 500-570

^d^ Blue = Percentage of PPF between 400-499 nm relative to total PPF

^e^ Green = Percentage of PPF between 500-599 nm relative to total PPF

^f^ Red = Percentage of PPF between 600-700 nm relative to total PPF

^g^ PPF reduction = Percent reduction in PPF relative to full sun

^h^ Data are presented as averages acquired on three different days: 12 June, 13 June, and 14 June 2020. Means are only compared within column and were separated with Fisher’s LSD. Means followed by a common letter are not significantly different (*P* = 0.05).

**Supplemental Table S12:** The effects of metal halide (MH) lamps on LEE Filters spectral quality.

| Filter(s) | R:FR | PPE | B:G | Blue ^d^ | Green ^e^ | Red ^f^ | PPF reduction ^g^ |
| --- | --- | --- | --- | --- | --- | --- | --- |
|  |  |  |  | % | | | |
| Lamps only | 1.44 A ^h^ | 0.80 A | 0.55 C | 24.0 D | 63.0 E | 13.0 A | - |
| 0.60 ND | 0.50 D | 0.73 C | 0.52 D | 22.0 E | 65.0 D | 13.0 A | 75.3 D |
| 1/2 CTB | 0.72 C | 0.74 B | 0.82 A | 34.3 A | 56.7 G | 9.0 D | 40.3 E |
| 1/2 PG | 1.12 B | 0.80 A | 0.38 E | 18.7 F | 69.3 B | 12.0 B | 23.3 F |
|  |  |  |  |  |  |  |  |
| 0.60 ND+  1/2 CTB | 0.26 F | 0.65 E | 0.77 B | 31.3 B | 59.0 F | 9.7 C | 86.0 B |
|  |  |  |  |  |  |  |  |
| 0.60 ND+  1/2 PG | 0.37 E | 0.72 D | 0.36 F | 17.7 G | 70.3 A | 12.0 B | 81.0 C |
|  |  |  |  |  |  |  |  |
| 0.60 ND+  1/2 PG+  1/2 CTB | 0.21 G | 0.64 F | 0.52 D | 24.7 C | 66.0 C | 9.3 CD | 89.0 A |

^a^ R:FR = 655-665 / 725-735

^b^ PPE = Phytochrome photoequilibria

^c^ B:G = 420-490 / 500-570

^d^ Blue = Percentage of PPF between 400-499 nm relative to total PPF

^e^ Green = Percentage of PPF between 500-599 nm relative to total PPF

^f^ Red = Percentage of PPF between 600-700 nm relative to total PPF

^g^ PPF reduction = Percent reduction in PPF relative to full sun

^h^ Data are presented as averages acquired on three different days: 12 June, 13 June, and 14 June 2020. Means are only compared within column and were separated with Fisher’s LSD. Means followed by a common letter are not significantly different (*P* = 0.05).
